# Ref-1 redox activity alters cancer cell metabolism in pancreatic cancer: exploiting this novel finding as a potential target

**DOI:** 10.1186/s13046-021-02046-x

**Published:** 2021-08-10

**Authors:** Silpa Gampala, Fenil Shah, Xiaoyu Lu, Hye-ran Moon, Olivia Babb, Nikkitha Umesh Ganesh, George Sandusky, Emily Hulsey, Lee Armstrong, Amber L. Mosely, Bumsoo Han, Mircea Ivan, Jing-Ruey Joanna Yeh, Mark R. Kelley, Chi Zhang, Melissa L. Fishel

**Affiliations:** 1 Department of Pediatrics and Herman B Wells Center for Pediatric Research, Indiana University School of Medicine, Indianapolis, IN 46202 USA; 2 Department of Medical and Molecular Genetics, Indiana University School of Medicine, Indianapolis, IN 46202 USA; 3 Department of Biohealth Informatics, IUPUI, Indianapolis, IN 46202 USA; 4 School of Mechanical Engineering, Purdue University, West Lafayette, IN 47906 USA; 5 Cardiovascular Research Center, Massachusetts General Hospital, Charlestown, MA 02115 USA; 6 Department of Medicine, Harvard Medical School, Boston, MA 02115 USA; 7 Department of Pathology and Laboratory Medicine, Indiana University School of Medicine , Indianapolis, IN 46202 USA; 8 Indiana University Simon Comprehensive Cancer Center, Indiana University School of Medicine, 1044 W Walnut St. R4-321, Indianapolis, IN 46202 USA; 9 Department of Biochemistry and Molecular Biology, Indiana University School of Medicine, Indianapolis, IN 46202 USA; 10 Purdue Center for Cancer Research, Purdue University, West Lafayette, IN 47906 USA; 11 Department of Microbiology and Immunology, Indiana University School of Medicine, Indianapolis, IN 46202 USA; 12 Department of Pharmacology and Toxicology, Indiana University School of Medicine, Indianapolis, IN 46202 USA

**Keywords:** scRNA-seq, Ref-1, Redox function, Metabolism, OXPHOS, Cancer associated fibroblasts (CAFs), Pancreatic cancer, Mitochondria

## Abstract

**Background:**

Pancreatic cancer is a complex disease with a desmoplastic stroma, extreme hypoxia, and inherent resistance to therapy. Understanding the signaling and adaptive response of such an aggressive cancer is key to making advances in therapeutic efficacy. Redox factor-1 (Ref-1), a redox signaling protein, regulates the conversion of several transcription factors (TFs), including HIF-1α, STAT3 and NFκB from an oxidized to reduced state leading to enhancement of their DNA binding. In our previously published work, knockdown of Ref-1 under normoxia resulted in altered gene expression patterns on pathways including EIF2, protein kinase A, and mTOR. In this study, single cell RNA sequencing (scRNA-seq) and proteomics were used to explore the effects of Ref-1 on metabolic pathways under hypoxia.

**Methods:**

scRNA-seq comparing pancreatic cancer cells expressing less than 20% of the Ref-1 protein was analyzed using left truncated mixture Gaussian model and validated using proteomics and qRT-PCR. The identified Ref-1’s role in mitochondrial function was confirmed using mitochondrial function assays, qRT-PCR, western blotting and NADP assay. Further, the effect of Ref-1 redox function inhibition against pancreatic cancer metabolism was assayed using 3D co-culture in vitro and xenograft studies in vivo.

**Results:**

Distinct transcriptional variation in central metabolism, cell cycle, apoptosis, immune response, and genes downstream of a series of signaling pathways and transcriptional regulatory factors were identified in Ref-1 knockdown vs Scrambled control from the scRNA-seq data. Mitochondrial DEG subsets downregulated with Ref-1 knockdown were significantly reduced following Ref-1 redox inhibition and more dramatically in combination with Devimistat in vitro. Mitochondrial function assays demonstrated that Ref-1 knockdown and Ref-1 redox signaling inhibition decreased utilization of TCA cycle substrates and slowed the growth of pancreatic cancer co-culture spheroids. In Ref-1 knockdown cells, a higher flux rate of NADP + consuming reactions was observed suggesting the less availability of NADP + and a higher level of oxidative stress in these cells. In vivo xenograft studies demonstrated that tumor reduction was potent with Ref-1 redox inhibitor similar to Devimistat.

**Conclusion:**

Ref-1 redox signaling inhibition conclusively alters cancer cell metabolism by causing TCA cycle dysfunction while also reducing the pancreatic tumor growth in vitro as well as in vivo.

**Supplementary Information:**

The online version contains supplementary material available at 10.1186/s13046-021-02046-x.

## Background

Pancreatic Ductal Adenocarcinoma (PDAC) is a devastating disease that has a 5-year survival rate of ~ 10% [1]. Associated with the actual tumor epithelial cell is a complex stroma composed of cancer-associated fibroblasts (CAFs), immune and endothelial cells, and a rich extracellular matrix (ECM) [2, 3]. It is predominantly characterized by severe hypoxia, a complex tumor micro-environment (TME) and altered metabolism that favors anabolic mechanisms [4–6]. PDAC cells exhibit altered cell metabolism to meet their bioenergetic, biosynthetic and reduction‐oxidation (redox) needs [7]. For example, one of the above mentioned anabolic pathways is pentose phosphate pathway, which supplies intermediates for nucleotide biosynthesis [8]. Other biomolecules like glutamine and aspartate which are necessary building blocks for tumor biomass are accumulated from the byproducts of the tricarboxylic acid (TCA) cycle [9, 10].

The regulation of many tumor suppressor genes and oncogenes is rewired to facilitate these new demands of the growing tumor. Genes such as TP53, KRAS and MYC can all be involved in the rewiring of the tumor’s metabolic capacity [8]. Smolkova et al., demonstrated four ‘waves’ of gene expression changes during carcinogenesis that involve reversible shifting between glycolysis and OXPHOS (oxidative phosphorylation) with the assistance of oncogenic signaling and mitochondrial gene reprogramming. These changes are the major alterations responsible for tumor initiation and metastasis [11]. The majority of PDAC cells harbor KRAS mutations which further enable tumor cells to survive under stress conditions like the lack of oxygen through stabilization of Hypoxia Inducible Factors (HIFs). Stabilized HIFs shift the balance between glycolysis and OXPHOS further away from OXPHOS towards glycolysis, partly by decreasing the activity of pyruvate dehydrogenase (PDH) [12–14].

Redox factor 1/Apurinic-apyrimidinic endonuclease 1 (Ref-1/APE1) (henceforth referred to as Ref-1) is a protein potentially impacting mitochondrial function through two of its major functions. Ref-1’s function as an endonuclease in base excision repair (BER) pathway could impact mitochondria as it repairs damaged mitochondrial DNA [15–18]. Ref-1 also possesses redox signaling promoting the binding of transcription factors (TFs) such as HIF-1α, STAT3, NF-κB, and AP-1, among others to DNA [19–24]. Ref-1’s regulation of HIF-1α along with other TFs may contribute to the metabolic rewiring that is observed in many cancers [24, 25]. As HIF stabilization would shift the cells toward glycolysis and away from oxygen-requiring OXPHOS, blockade of Ref-1 under hypoxia leads to a downregulation of glycolysis as HIF1 cannot be fully activated when Ref-1 is blocked. Surprisingly, we also observed dramatic gene expression changes in the TCA cycle genes as well as genes within the complexes of the electron transport chain (ETC). There are reports in cancer including PDAC that depending on the glucose and oxygen conditions that OXPHOS can be utilitized especially under extreme oxygen conditions [26, 27]. These effects can be driven by ROS and even Akt and NF-κB signaling pathways and impact upon metabolism [28]. NF-κB is also under Ref-1 redox activation control. Multiple cancers including PDAC exhibit increased Ref-1 expression levels that concomitantly associate with their resistance to radiation and chemotherapy and potentially to a metabolic shift, leading to poorer patient prognosis [29, 30].

Due to Ref-1’s role in regulating multiple TFs associated with cancer-related pathways, Ref-1 has become a novel target for anti-cancer therapy in PDAC [25, 31, 32]. Although the redox signaling functions of Ref-1 have been studied in a number of biological contexts, its interacting TF partner(s) critical for regulation of metabolic pathways in PDAC is still not well understood. We previously published single-cell RNA sequencing (scRNA-seq) data in low passage patient-derived PDAC cells following Ref-1 knockdown under normoxia [25]. In order to better understand the role of Ref-1 on hypoxia signaling in PDAC, we used scRNA-seq coupled with proteomics to further investigate the role of Ref-1 under hypoxic conditions. In our current study, bioinformatic analysis of hypoxia scRNA-seq data demonstrated that knockdown of Ref-1 led to distinct variation in metabolic pathways including TCA cycle (top downregulated pathway), glycolysis and OXPHOS, HIF regulated genes, apoptosis, immune response, and a series of signaling pathways and transcriptional regulatory factors. In addition to the expected decrease in glycolysis, dramatic decreases in gene expression in TCA cycle, complexes within the electron transport chain and less availability of NADP + resulting in higher levels of oxidative stress were also observed. All of these are novel findings. Using Ref-1 siRNA and specific Ref-1 redox inhibitors, we validated these findings using qRT-PCR on a panel of mitochondrial differentially expressed genes (DEGs) and quantitation of mitochondrial substrates. We also extended these studies to cancer-associated fibroblasts (CAFs) which constitute a crucial component of the PDAC TME. In order to ascertain the clinical relevance of our findings, we performed in vivo studies using mice with tumor cells co-injected with CAFs treated with Ref-1 inhibitor, APX2009 or the comparator compound, Devimistat, also known as CPI-613. APX2009 is a second-generation Ref-1 redox signaling inhibitor following on APX3330 which successfully completed a phase I clinical trial in solid tumors [33–36]. Devimistat is a first-in-class dehydrogenase inhibitor targeting the TCA cycle [37–39]. In summary, our findings demonstrate a newly discovered role for Ref-1 in TCA cycle and expression of genes within the mitochondrial complexes even under hypoxia as well as confirm that Ref-1 redox function has a role in regulating PDAC hypoxia signaling pathways as HIF regulated genes and glycolysis were also affected. Additionally, we identified a potential PDAC therapeutic approach using APX2009 and Devimistat in multiple 3D co-culture models containing both pancreatic cancer cells as well as CAFs which was also validated in vivo.

## Methods

### Cell culture

Pa03C, Panc10.05, and CAF19 cells were obtained from Dr. Anirban Maitra at The Johns Hopkins University [40] and were then transduced with TdTomato for tumor cells or EGFP for CAFs as previously described [41]. CAF02-hTERT cells were isolated using the outgrowth method as previously described [42, 43]. 143B cybrid pairs (143B-wt and 143B-CytB) were gifted from Dr. M.G. Vander Heiden (Massachusetts Institute of Technology) with the permission of Dr. N.S. Chandel (Northwestern University) and were cultured in the standard medium consisting of DMEM (Invitrogen #11,965) supplemented with 10% fetal bovine serum, 1 mM of sodium pyruvate, 1% penicillin–streptomycin and 0.1 mg/mL uridine at 37 ºC with 10% CO_2_. Phenformin (14,997) was purchased from Cayman Chemical Company. Cells were maintained at 37 °C in 5% CO2 and grown in DMEM (Invitrogen; Carlsbad, CA) with 10% Serum (Hyclone; Logan, UT), or under hypoxic conditions of 1% O_2_ / 5% CO_2_ using a Ruskinn Invivo_2_ 200 hypoxia work station. Cell line identity was confirmed by DNA fingerprint analysis (IDEXX BioResearch, Columbia, MO) for species and baseline short-tandem repeat analysis testing. Cell lines were 100% human and a nine-marker short tandem repeat analysis exists on file. They were also confirmed to be mycoplasma free.

### Single cell RNA sequencing (scRNA-seq)

scRNA-seq was performed as previously reported [25]. Libraries were prepared by The Purdue Genomics Facility (Purdue University, West Lafayette, IN) using a Nextera kit (Illumina, San Diego, CA, USA). Unstranded 2 × 100 bp reads were sequenced using the HiSeq2500 (Illumina, San Diego, CA, USA) on rapid run mode in one lane.

### scRNA-seq data analysis

FastQC was applied to evaluate the quality of the single cell RNA sequencing data. Counts were called for each cell sample by using STAR alignment pipeline against human GRCh38 reference genome. Cells with less than 250 or more than 10,000 non-zero expressed genes were excluded from the analysis. Cells with more than 15% counts mapped to the mitochondrial genome were excluded as low quality cells, resulting in 40 Ref-1 knockdown (KD) and 48 Scr cells under hypoxia condition and 27 Ref-1 KD and 46 Scr cells under normoxia condition for further analysis.

Unsupervised cell clustering was performed using Seurat v3.0 with the variedly expressed genes identified by default parameters. Cell clusters were annotated by the experimental condition and their uniquely highly expressed genes. Differentially expressed genes and gene expression states were identified by using the left truncated mixture Gaussian model based test, with FDR < 0.05 as the significant cutoff [44]. Gene co-regulation modules were identified by using our inhouse developed Boolean matrix decomposition method MEBF [45]. Pathway enrichment of the differentially expressed genes and the genes in each co-regulation module were analyzed by a hypergeometric test against the canonical gene sets and transcriptional regulatory factor targets retrieved from MsigDB v6 [46], with *p* < 0.001 as the significant cutoff.

### Quantitative global proteomic comparison of protein levels

Sample preparation, mass spectrometry analysis, bioinformatics and data evaluation were performed in collaboration with the Proteomics Core Facility (Indiana University School of Medicine, Indianapolis, IN). Detailed method can be found in supplementary information (Supplementary Table 1) and was adapted from literature reports [47, 48] and vendor provided protocols. Briefly, cells were lysed using urea lysis buffer. Protein isolated and trypsin/Lys-C digested for peptides. Peptides were fractionated using Pierce™ High pH reversed-phase peptide fractionation spin columns and then subjected to Nano-LC–MS/MS analysis.

### Transfection with Ref-1 and scrambled siRNA

The siRNAs used were: Scrambled (Scr) (5′-CCAUGAGGUCAGCAUGGUCUG-3′, 5′- GACCAUGCUGACCUCAUGGAA-3′) and siRef-1 (5′-GUCUGGUACGACUGGAGUACC-3′, 5′-UACUCCAGUCGUACCAGACCU-3′). All siRNA transfections were performed as previously described [41, 49–52]. Briefly, 1 × 10^5^ cells are plated per well of a 6-well plate and allowed to attach overnight. The next day, Lipofectamine RNAiMAX reagent (Invitrogen, Carlsbad, CA) was used to transfect in the Ref-1 and Scr siRNA at concentrations of 10 nM following the manufacturer’s indicated protocol. Opti-MEM, siRNA, and Lipofectamine was left on the cells for 16 h and then regular DMEM media with 10% Serum was added. Cells were assayed for RNA and protein expression 3 days following transfection.

### Inhibitor treatment

APX3330 and APX2009 were provided by Apexian Pharmaceuticals (20 N. Meridian, Indianapolis, IN 46204, USA) and prepared as previously described [35, 36, 53]. Devimistat (CPI-613) was purchased from APExBio (Boston, MA) and reconstituted in 100% DMSO as a 50 mM stock for in vitro drug treatments. Both agents are in clinical trials (clinicaltrials.gov; NCT03375086 and NCT03435289 respectively).

Pa03C cells (2 × 10^5^) were plated into 6-well plates and allowed to attach overnight. The following day, APX3330, APX2009, or Devimistat in low serum (5%) DMEM media were added to the wells. DMSO was used as the vehicle control. Cells were treated for 24 h, after which they were collected for analysis.

### Mitochondrial function assay

S-1 Mitoplates (Biolog, Hayward, CA) were used to investigate mitochondrial function. Assays were performed as per manufacturer’s protocol. Briefly, plates were activated by adding the Assay Mix to the wells to dissolve the substrates, for at least 60 min at 37 °C. Following siRef-1 transfection or drug treatment, cells were collected, counted, resuspended in provided buffer and plated at 5 × 10^4^ cells/well. This resuspension was added to the plate, which was immediately read at 590 nm kinetically at 5 min intervals for 4 h at 37 °C. Data was analyzed using Graphpad Prism 8, and statistical significance was determined using the 2-way ANOVA and *p*-values < 0.05 were considered statistically significant.

### Western blot analysis

For whole cell lysates, cells were harvested, lysed in RIPA buffer (Santa Cruz Biotechnology, Santa Cruz, CA), and protein was quantified and electrophoresed. Immunoblotting was performed using the following antibodies: Ref-1 (1:1000, Novus Biologicals, Littleton, CO) and Vinculin (1:1000, Sigma, St. Louis, MO). For subsequent experiments, Ref-1 expression needed to be decreased by at least 80% compared to scrambled control in order to be considered for further analysis.

### qRT-PCR

qRT-PCR was used to measure the mRNA expression levels of the various genes identified from the scRNA-seq analysis. Following transfection, total RNA was extracted from cells using the Qiagen RNeasy Mini kit (Qiagen, Valencia, CA) according to the manufacturer’s instructions. First-strand cDNA was obtained from RNA using random hexamers and MultiScribe reverse transcriptase (Applied Biosystems, Foster City, CA). Quantitative PCR was performed using SYBR Green Real Time PCR master mix (Applied Biosystems, Foster City, CA) in a CFX96 Real Time detection system (Bio-Rad, Hercules, CA). The relative quantitative mRNA level was determined using the comparative Ct method using actin as the reference gene. The primers used for qRT-PCR are detailed in Supplementary Table 2 (Glycolysis, TCA Cycle, OXPHOS and other genes). Experiments were performed in at least triplicate for each sample. Statistical analysis performed using the 2^−ΔΔ^*C*_*T*_ method and analysis of covariance (ANCOVA) models, as previously published [23].

### NADP-NADPH assay

NADP/NADPH Assay Kit from Abcam (ab65349) was used to measure the ratio of NADPH to NADP using Pa03C cells. Assay was performed as per manufacturer’s protocol. Briefly, cytoplasmic NADPH/NADP was extracted from 4 × 10^6^ cells after treatment with Vehicle control or APX2009 or RN7-58 in a time course using 400 μL extraction buffer provided by the manufacturer. Samples were sheared and passed through DNA spin columns. For NADPH detection, 150 μL of extracted samples were heated to 60 °C for 30 min to decompose NADP and the remaining sample for total NADP (NADPt). 100 μL of Reaction Mix was added to 50 μL of standard or sample/well and incubated for 5 min at room temperature. 10 μL of NADPH Developer was added into each well. Multiple readings at OD450nm were taken during 1–8 h. NADP + /NADPH was calculated as NADPH/NADP + ratio = NADPH/(NADPt—NADPH). The measured NADP and NADPH levels were calculated by comparison with a standard curve.

### In vivo studies

All animal studies were conducted under the guidelines of the National Institutes of Health and were approved by the Institutional Animal Care and Use Committee of Indiana University School of Medicine. NOD.Cg-Prkdcscid Il2rgtm1Wjl/SzJ (NOD/SCIDγ(-/-))mice (or other strains) were obtained from the In Vivo Therapeutics Core of the Indiana University Simon Cancer Center. Animals were maintained under pathogen-free conditions and a 12 h light–dark cycle. NOD scid gamma (NOD.Cg-*Prkdc*^*scid*^* Il2rg*^*tm1Wjl*^/SzJ) or NSG mice were subcutaneously implanted with Pa03C cells (2.5 × 10^6^) or with Panc10.05 (5 × 10^6^) + CAF19 (15 × 10^6^) cells in the hind flank using a 200 μl volume of 50:50 solution of Matrigel:DMEM medium. When tumor volumes reached ~ 100 mm^3^, the mice were randomized into 4 groups of 8–9 mice before commencing treatment (~ 11 days for Pa03C and ~ 24 days for Panc10.05 + CAF19 post implantation). The treatment regimen consisted of oral administration of either vehicle (Propylene Glycol, Kolliphor HS15, Tween 80 (PKT) as previously reported [34]) or 35 mg/kg APX2009 or 50 mg/kg Devimistat dosed twice a day for 15 (for Pa03C xenografts) or 20 (for Panc10.05 + CAF19 xenografts) days. Tumor volumes were measured twice a week and mice were weighed once a week. Data was analyzed using Graphpad Prism 8. Statistical significance was determined using the one-way ANOVA and *p*-values < 0.05 were considered statistically significant.

### 3D co-culture assays

Ultra-low attachment 96-well plates (Corning Inc., Life Sciences) were used to generate 3-dimensional tumor spheroids in the presence of CAFs as reported in the Results and as described previously [41, 54, 55]. Following plating, cells were treated on Days 4, 7, and 10 with media containing 5% serum, 3% growth factor reduced Matrigel, and inhibitors as indicated. On Days 4, 7, 10, and 14, spheroids were analyzed using Thermo ArrayScan high-content imaging system [56]. Images of 3D structures were captured by ArrayScan using a 2.5 × objective for TdTomato and EGFP; then 2D projections were processed to quantify differences in total intensity of both CAFs and tumor.

### Interstitial tumor-microenvironment-on-chip (iT-MOC) assay

The interstitial tumor-microenvironment-on-chip (iT-MOC) is a 3D in vitro microfluidic platform having two layers of microchannels interfaced with a porous membrane in between. Details of fabrication and preparation were described previously [57–59]. Briefly, pancreatic cancer cells (Panc10.05) and cancer-associated fibroblasts (CAF19) were mixed at a 1:1 cell ratio into the cell-collagen mixture. The initial cell concentration was 2 × 10^6^ cells/mL for each cell type. After loading, the devices were incubated at 37 °C for 1 h for collagen gelation. Then, the culture medium was perfused by pressurizing the interstitial channel.

In order to assess drug efficacy, the PDAC iT-MOC was cultured for 48 h before treatment. On day 2, drugs were perfused through the capillary channel. The drug solutions were prepared as 0 µM (control), 30 µM of APX2009, 25 µM of Devimistat, and the combination of APX2009 (30 µM) and Devimistat (25 µM) in the culture medium. On day 5, the channels were washed with drug-free medium and re-perfused with the same drug-containing medium. On day 8, the iT-MOC platforms were washed and cultured in normal culture medium for 24 h before the viability assay. Drug efficacy was analyzed in two ways, cell growth and cell survival as detailed in the supplementary methods.

### Statistics

All the experiments were performed at least three independent times. The data obtained were expressed as ‘Mean + Standard Error’. Significance was calculated as per either 2-way ANOVA or unpaired t-test wherever applicable using Graph Pad Prism Version 8. For iT-MOC assays, Drug efficacy differences of each drug control were statistically analyzed by Tukey post hoc multiple comparison test provided in ANOVA. The difference was considered statistically significant when *p*-value < 0.05. For qRT-PCR, analysis of covariance models was performed to test the Ct difference of each target gene value between treatment with APX2009, Devimistat (CPI-613), and vehicle (DMSO) or siRNA and scrambled control after standardization by reference gene (RPL6/Actin) using ANCOVA as previously described [23]. A p-value of at least < 0.05 was considered statistically significant.

To test the tumor growth rate following treatment (i.e., the regression slope for a particular treatment) and differences in tumor growth rates between treatments (i.e., the difference in regression slopes between two treatments) in the in vivo tumor model, mixed effect repeated measure regression models with random intercept were used [60]. Tumor weights over time were estimated and compared between treatments from the regression models. To be considered statistically significant, a p-value of at least 0.05 was used. All statistical analysis was conducted using SAS 9.4 (SAS, Inc., Cary, NC, 2016).

## Results

### Transcriptomic variation caused by Ref-1 inhibition under hypoxia

A scRNA-seq experiment was conducted on a patient-derived pancreatic cancer line, Pa03C under the following conditions: Ref-1 knockdown (siRef-1), Scrambled control (Scr), Hypoxia (H), and Normoxia (N). The Fluidigm C1 Smart Seq2 protocol was used for the scRNA-seq as described in Methods. This platform enabled a saturated measure of the transcriptome in each single cell, resulting in high sensitivity in the identification of the genes, biological pathways or transcriptional regulatory modules regulated by Ref-1 [61, 62]. In total, 40 cells transfected with Ref-1 siRNA and 48 Scr control cells were collected after hypoxia exposure and after removing low quality cells. We combined this dataset with 27 cells of Ref-1 siRNA and 25 Scr control collected under normoxia from our previous work. In total, 18,204 genes with a significant none zero expression were detected in this data set. It is noteworthy the major goal of this study was not for cell type identification. Instead, we focused on identification of the distinct functional changes that are due to the inhibition of Ref-1 and/or perturbed oxygen level, i.e. biologically explainable gene sets that show distinct expression variation in a subset of the sample. Our previous studies demonstrated these cell numbers provide enough statistical power for the analysis of differentially expressed genes and functional modules [44, 63].

**Fig. 1:**
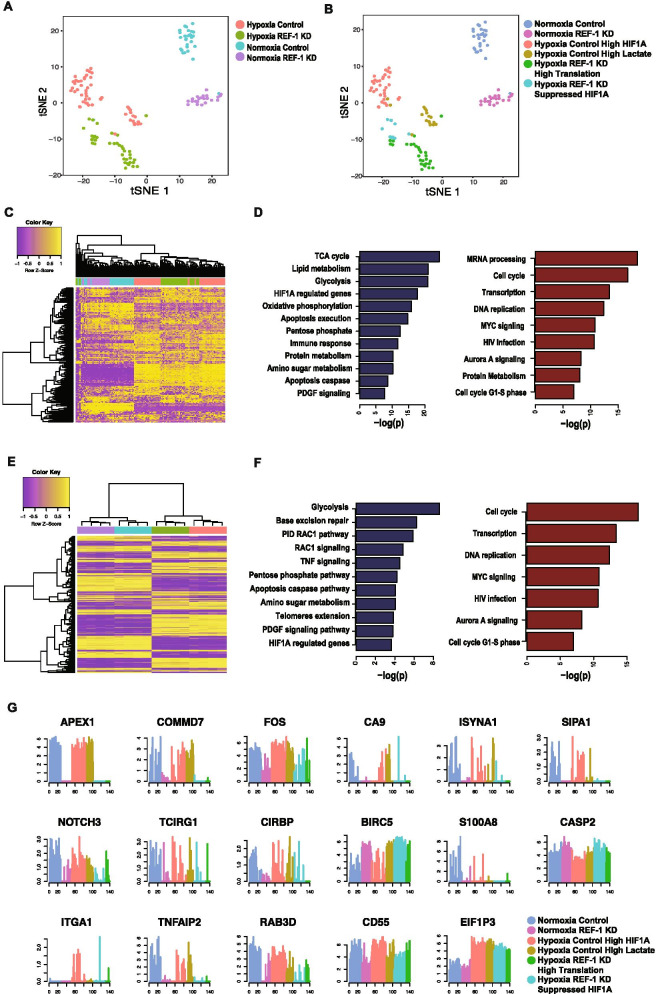
Integration of scRNA seq and proteomics following Ref-1 downregulation under hypoxia. TSNE plot of the scRNA-seq data colored by experimental conditions (**A**) and inferred cell clusters from unsupervised cell clustering (**B**). **C** Heatmap of the top 250 genes with largest dispersion in the scRNA-seq data. High and low expression are colored by yellow and purple, respectively. The column color code represents the experimental condition of each cell annotated in (**A**). **D** The top pathways in scRNA-seq data enriched by the down- (blue) and up- (red) regulated genes in Ref-1 KD vs Scr control under hypoxia. The x-axis is -log(p.value) assessed by hypergeometric test. **E** Heatmap of the proteomics data with the same color code as in (**C**). **F** The top pathways observed in the proteomics data enriched by the down- (blue) and up- (red) regulated proteins in siRef-1 vs Scr control under hypoxia. **G** Gene expression profile of selected genes with significant differential expression through the cell clusters. The color code is the same as the cell clusters annotated in (**B**)

The expression level of Ref-1 (APEX1) in the single cells demonstrated the gene was successfully knocked down in the siRef-1 group (Fig. 1G). Unsupervised cell clustering analysis was first conducted on the cells under all conditions (See Methods). Six cell clusters were identified, which were all highly associated with the experimental groups (Fig. 1A, B). Genes overly expressed in each cluster were identified by using left truncated mixture Gaussian model. The cell clusters were further annotated by the experimental condition and their specifically expressed genes and pathways. Two clusters within the hypoxia Scr control cells were identified. Pathway enrichment analysis of the marker genes of each cluster suggested one group has high expression of HIF-1α-regulated genes while the other has specifically elevated lactate production. Within the siRef-1 cells under hypoxia, two clusters were identified; one cluster corresponded to consistently down-regulated genes that were downstream of HIF-1α which would be expected based on Ref-1 redox regulation of HIF-1α. The other cluster corresponded to genes related to upregulated translation (Fig. 1B).

Figure 1C illustrates the heatmap of the top variably expressed genes in all the tumor cells. Distinct clusters of the cells associated with different conditions were observed. Dendrogram derived by a hierarchical clustering analysis demonstrated dramatic changes in gene expression caused by hypoxia. DEGs between siRef-1 and Scr control were identified using left truncated mixture Gaussian model [44]. In hypoxia vs normoxia cells, we have identified 3,521 upregulated and 472 downregulated genes (FDR < 0.05). Similarly, there are significant changes in gene expression upon comparison of siRef-1 and scrambled control under the hypoxia condition, where 386 upregulated and 1,251 downregulated genes were identified. Matched samples were also collected for bulk proteomic analysis of the four conditions, totaling 6,931 proteins measured. Proteomic analysis confirmed significant differences between hypoxia and normoxia (2,155 upregulated and 1,825 downregulated proteins, *p* < 0.01 by Mann Whitney test) as well as siRef-1 and scrambled control under hypoxia condition (513 upregulated and 390 downregulated proteins, *p* < 0.01 by Mann Whitney test). Complete lists of the dysregulated genes and proteins were provided in Supplementary Table S3.

To characterize the Ref-1-regulated genes under hypoxia, we focused on the 386 upregulated and 1,251 downregulated genes in siRef-1 vs Scr control under 1% O_2_ levels. Pathway analysis suggested the upregulated genes significantly (*p* < 0.001) enrich mRNA processing and transcription, cell cycle, DNA replication, MYC signaling, HIV infection, and protein metabolism. PDAC cells with lower Ref-1 expression proliferate much slower compared to Scr control and the cell cycle genes that were upregulated were mainly nucleoporins and some proteosome genes, but not cyclins. The downregulated genes mainly (*p* < 0.001) enrich in central metabolic pathways (glycolysis, TCA cycle, pentose phosphate, oxidative phosphorylation, amino acids, protein and lipid metabolism), with HIF-1α and PDGF signaling pathways also affected (Fig. 1D). For comparison under normoxia, there were also more genes downregulated than upregulated identified in siRef-1 vs Scr control (447 vs. 120, FDR < 0.05). Pathway enrichment analysis revealed the upregulated genes significantly (*p* < 0.001) enrich to transcription, HIV infection and immune response pathways, while the downregulated genes significantly (*p* < 0.001) enrich to central metabolic pathways (glycolysis, pentose phosphate, oxidative phosphorylation), immune response (antigen presentation, T cell receptor and IL6 signaling), and multiple signaling pathways (p53, MAPK, MET and HIF-1α). We also integrated the scRNA data analysis with the proteomic analysis and determined that the differentially expressed genes and pathways identified from the scRNA-seq data are highly consistent to the significant proteins observed in the proteomics data, especially for the upregulated cell cycle (nucleoporins) and transcription pathways and downregulated metabolic, apoptosis and signaling pathways under hypoxia condition (Fig. 1D,F). Figure 1G shows selected genes associated with each condition and specific cell clusters within the siRef-1 hypoxia group. Complete lists of the differentially expressed pathways were provided in Supplmentary Table S4. Interestingly, the top downregulated pathway under hypoxia was the TCA cycle with glycolysis and OXPHOS also in the top five.

One of Ref-1’s major functions is its redox activity in which Ref-1 converts an oxidized transcription factor into a reduced transcription factor which leads to an increase in its DNA binding and functional activity. We quantitated the activity of various transcription factors after Ref-1 knockdown by identifying the gene co-regulation modules that are associated with different experimental conditions, i.e. hypoxia [44, 64]. Specifically, gene-wise expression states were first inferred by the left truncated mixture Gaussian model. Modules of genes that show consistent activated or suppressed expression in a subset of cells were identified using a non-negative matrix factorization method namely MEBF [65]. We further evaluated the enrichment of the genes in each module against known targets of transcriptional regulatory factors, and the association of the cells of each module with the experimental conditions. Supplementary Table S5 lists the predicted transcriptional regulatory factors of each identified module (see details in Methods). Specifically, downregulation of the gene modules possibly regulated by CTCF, SP1, POLR2A, MAX, CEBPB, REST, MYC, JUN, JUND, NFKB1, STAT3, STAT1, HIF1A, FOXA1, and CREB1 were identified in siRef-1 vs Scr control under both hypoxia and normoxia condition, confirming interactions between Ref-1 and its known transcriptional regulators.

### Metabolic shifts following treatment with Ref-1 inhibitor observed from scRNA-seq data

Noting the scRNA-seq and proteomics data consistently revealed downregulated central metabolism pathways in siRef-1 vs Scr control under both hypoxia and normoxia, we specifically focused on characterizing the gene expression alterations in central metabolic pathways. These pathways included glycolysis, pentose phosphate pathway, lactate production, TCA cycle, oxidative phosphorylation, glutaminolysis, and other amino acid metabolism that can fuel the production of key metabolites succinate, fumarate, malate and oxaloacetate in the TCA cycle.

**Fig. 2:**
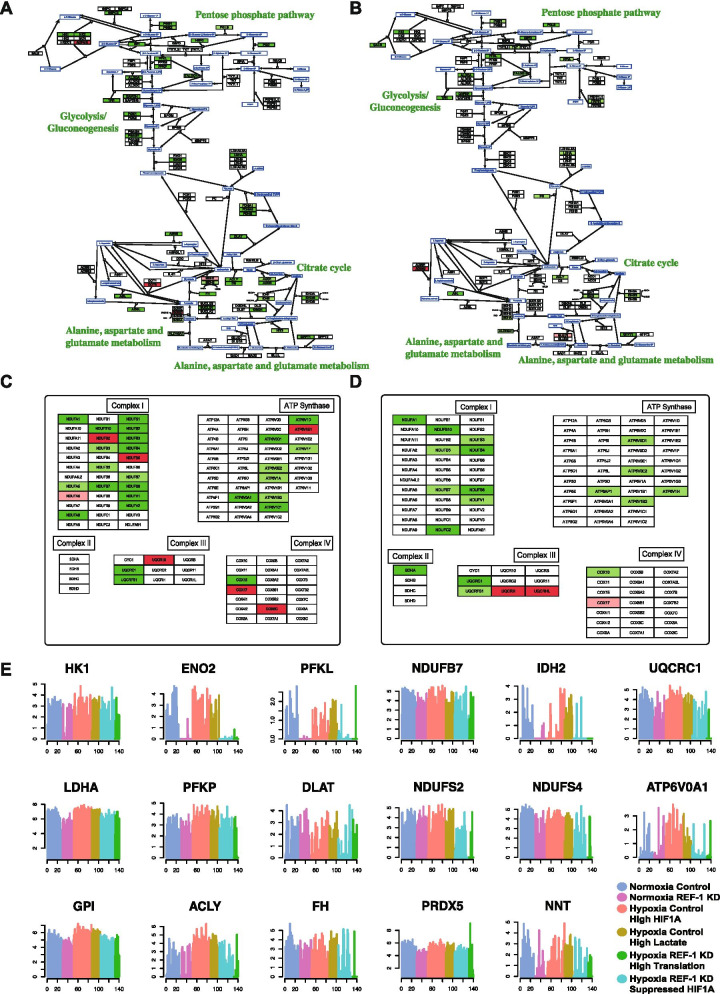
Blockade of Ref-1 under hypoxia causes downregulation of metabolic pathways. Differentially expressed central metabolic genes in siRef-1 vs Scr Control under hypoxia (**A**) and normoxia (**B**) conditions. The up- and down- regulated genes were colored by light (0.001 < *p* < 0.05) or dark (*p* < 0.001) red and green, respectively. Differentially expressed mitochondrial complex and ATP synthase genes in siRef-1 vs Scr control under hypoxia (**C**) and normoxia (**D**). **E** Gene expression profile of key genes involved in the central metabolism through different cell groups. The color code is the same as Fig. 1G

scRNA seq data presented in Fig. 2 illustrates the dysregulated enzymes involved in the selected central metabolic pathways in siRef-1 vs Scr control under hypoxia (Fig. 2A) and normoxia (Fig. 2B). Glycolysis (*p* = 5.7e-6), TCA cycle (*p* = 1.1e-7) and oxidative phosphorylation (*p* = 1.7e-8) pathways were significantly enriched in downregulated genes in siRef-1 vs Scr control under hypoxia. In addition, the enzymes phosphogluconate dehydrogenase (PGD) and transaldolase (TALDO1) that catalyze the first steps of glucose flow into pentose phosphate were significantly downregulated in siRef-1 vs Scr control. Significant downregulation of lactate dehydrogenase A (LDHA) was also observed. The enzymes asparagine synthetase (ASNS), argininosuccinate lyase (ASL), and adenylosuccinate lyase (ADSL) involved in the metabolism of aspartate to fumarate and oxaloacetate were also significantly downregulated. Under normoxia, the glycolysis (*p* = 7.5e-7) and oxidative phosphorylation (*p* = 6.2e-4) pathways were also significantly enriched in downregulated genes in siRef-1 vs Scr control but were not as dramatically reduced compared to hypoxia (Fig. 2A, B). Although the TCA cycle, lactate production, and aspartate metabolic genes are downregulated under normoxia, the effects are more robust under hypoxia, and no significant changes in genes involved in glutaminolysis were observed when Ref-1 levels are reduced under either hypoxia or normoxia.

With the reduction in expression of genes within glycolysis, TCA cycle, and oxidative phosphorylation pathways, genes involved in the mitochondrial respiratory complexes I-IV as well as ATP synthases were also investigated. Figure 2C and D show the dramatic downregulation of genes in the mitochondrial respiratory complexes I-IV and ATP synthases. When Ref-1 is knocked down and the cells are exposed to hypoxia, significant downregulation of the mitochondrial complex I (*p* = 0.001), complex III (*p* = 0.03) and ATP synthase (*p* = 0.0004) genes were observed. Similarly, statistically significant but less downregulated mitochondrial complex I (*p* = 0.009) genes were observed under normoxia. Figure 2E highlights the expression profile of selected genes involved in these central metabolic pathways under the conditions of hypoxia/normoxia and Ref-1 knockdown.

### Ref-1 inhibition directly effects genes important in glycolysis, TCA cycle, and OXPHOS complexs

**Fig. 3:**
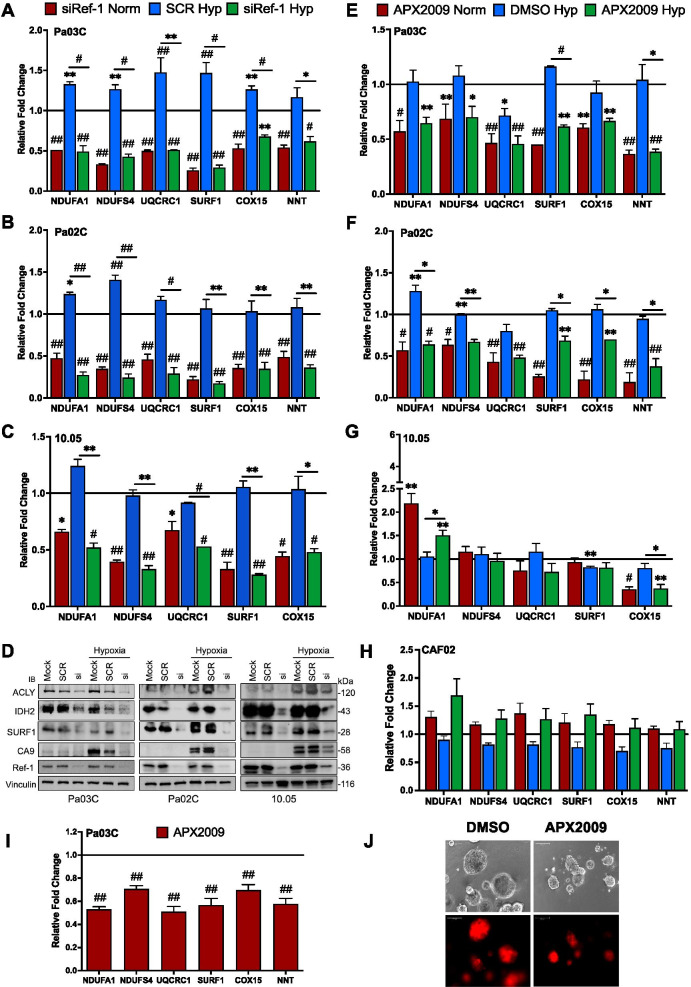
Ref-1 inhibition downregulates mitochondrial complex genes as well as Ref-1 PD marker genes. **A**-**C** Validation of selected mitochondrial complex genes from the scRNA-seq data using qRT-PCR in Pa03C (*n* = 3), Pa02C (*n* = 3), and Panc10.05 (*n* = 2) cells (Scr/siRef-1 – 30 nM, 1% hypoxia for 24 h, *p* < 0.05–0.0001). **D** Western Blots representing downregulation of mitochondrial metabolic proteins with Ref-1 knockdown 72 h post transfection. **E**–**G** Expression of mitochondrial complex genes after treatment with Ref-1 redox inhibitor (APX2009-10 µM for Pa03C, 15 µM for Pa02C and 20 µM for Panc10.05 cells for 28 h) under normoxia and hypoxia (1%O_2_ for 24 h) (*n* = 2, *p* < 0.05–0.0001). **H** Mitochondrial complex gene expression following Ref-1 redox inhibition (APX2009-10 µM 28 h) under normoxia and hypoxia (1%O_2_ for 24 h) in CAFs (*n* = 3). **I** Mitochondrial complex gene panel after treatment with Ref-1 redox inhibitor (APX2009, 5 µM) compared to vehicle control (DMSO) in Pa03C 3D spheroids (*n* = 3, *p* < 0.05–0.0001) and **J** Images representing the spheroids of Pa03C cells. (Scale Bar – 100 µm, 10X mag). Relative fold change refers to the gene expression changes when compared to Scr or vehicle-treated cells under normoxia

Following the scRNA-seq and proteomic bioinformatic analysis in Figs. 1 and 2, gene expression changes were validated in Pa03C, Pa02C, and Panc10.05 cells using a panel of genes that are involved in glycolysis, TCA cycle, and OXPHOS (within the various complexes of the ETC. Knockdown of Ref-1 significantly downregulated the expression of the panel of six genes belonging to OXPHOS complexes in all three cell lines, confirming the scRNA-seq data (Fig. 3A-C). These six genes were not strongly induced by hypoxia (~ 1.5-fold), but the downregulation was consistent when Ref-1 levels were reduced under both conditions. Using these same PDAC cell lines, 4 out of 5 genes within the glycolysis and TCA cycle pathways were downregulated with Ref-1 knockdown (Supplemental Fig. S1A-D). To further confirm the qPCR data at the protein level, western blotting demonstrated similar downregulation with Ref-1 knockdown for ATP citrate lyase (ACLY), isocitrate dehydrogenase (IDH2), and SURF1 under both normoxia and hypoxia (Fig. 3D and supplemental Fig. S1E-G). CA9 was used a positive control for hypoxia as well as a marker of Ref-1 inhibition as in our previous studies [41, 53]. In order to delineate which function of Ref-1 was driving the decrease in gene expression of the mitochondrial metabolic genes, cells were treated with APX2009, a second-generation Ref-1 redox specific inhibitor, which has no effect on Ref-1 DNA repair endonuclease activity [66]. All six genes related to OXPHOS were significantly reduced following Ref-1 redox inhibition in Pa03C and Pa02C cells grown in monolayer, however NDUFS4 was not as dramatic as the results with Ref-1 knockdown (Fig. 3E, F). Also, the decreases in expression of the OXPHOS gene panel were not as dramatic in Panc10.05 after treatment with APX2009 inhibition with the exception of COX15 (Fig. 3G). Genes within glycolysis and TCA cycle demonstrated similar reduction in expression with both Ref-1 siRNA and APX2009 treatment, with the exception of the SDHA gene (Supplemental Fig. S1H-M), As 3D spheroid cultures of PDAC cells mimic in vivo tumor hypoxic regions, further evaluation of these genes was done using Pa03C spheroids. Similar downregulation was observed for mitochondrial complex genes (Fig. 3I) as shown in Fig. 3E. Representative pictures of the 3D spheroids following treatment with vehicle (DMSO) or APX2009 are shown in Fig. 3J. In stark contrast, cancer-associated fibroblast (CAF) cells that would be found within the tumor microenvironment did not demonstrate the same changes in gene expression of these mitochondrial genes as was observed in the tumor cells under either hypoxia or normoxia (Fig. 3H).

Using an established panel of twelve genes that were significantly changed with Ref-1 siRNA treatment under normoxia, we evaluated the expression of these genes as markers of Ref-1 inhibition under conditions of hypoxia and treatment with Ref-1 redox signaling inhibitor, APX2009 [25]. Under conditions of hypoxia, expression of CIRBP, ITGA1, NOTCH3, TAPBP, and CA9 was upregulated and then significantly reduced following Ref-1 redox inhibition with APX2009 with the exception of ITGA1 (Supplemental Fig. S3N). Expression of ABCG2, COMMD7, ISYNA1, RAB3D, SIPA1, TAPBP, TNFAIP2, and BIRC5 was significantly reduced following Ref-1 redox inhibition with APX2009 regardless of the oxygen conditions. PPIF was downregulated under hypoxia and was upregulated with Ref-1 inhibition under normoxia as well as hypoxia. Two genes, ABCG2 and ITGA1 went opposite to that observed with Ref-1 knockdown [25] (ABCG2 was downregulated and ITGA1 was upregulated with APX2009 treatment, Fig. S3N). BIRC5 (Survivin) and CA9 were used as controls and were previously published as markers of Ref-1 redox inhibition (Supplemental Fig. S3N) [21, 41].

Again using 3D spheroid cultures of PDAC cells, further evaluation of these Ref-1 regulated genes was done. Similar yet more pronounced downregulation was observed for Ref-1-regulated gene subsets, with the exception of NOTCH3 (Supplemental Fig. S3O). As seen previously, PPIF gene expression was significantly upregulated with Ref-1 redox inhibition using APX2009.

### Functional validation of the defect in metabolic pathways following Ref-1 knockdown shows reduced TCA cycle substrates

The effect of glucose levels in the growth conditions of both Scr control and siRef-1 was tested using 3D spheroid assays incorporating both patient-derived tumor cells as well as CAFs (Supplemental Fig. S2). Pa03C cells were transfected with Ref-1 or Scr siRNA and cultured with or without CAFs in regular or low-glucose (LG) media. In presence and absence of CAFs, the Scr control spheroids grew significantly slower in the LG media compared to regular growth media. The cultures containing CAFs were less affected by the low glucose media, suggesting that the CAFs are providing that necessary tumor growth support (Supplemental Fig. S2B). However, when Ref-1 was knocked down, there was no difference in growth regardless of the media, further demonstrating that the main effect on growth of the spheroids was the lack of Ref-1 expression.

**Fig. 4:**
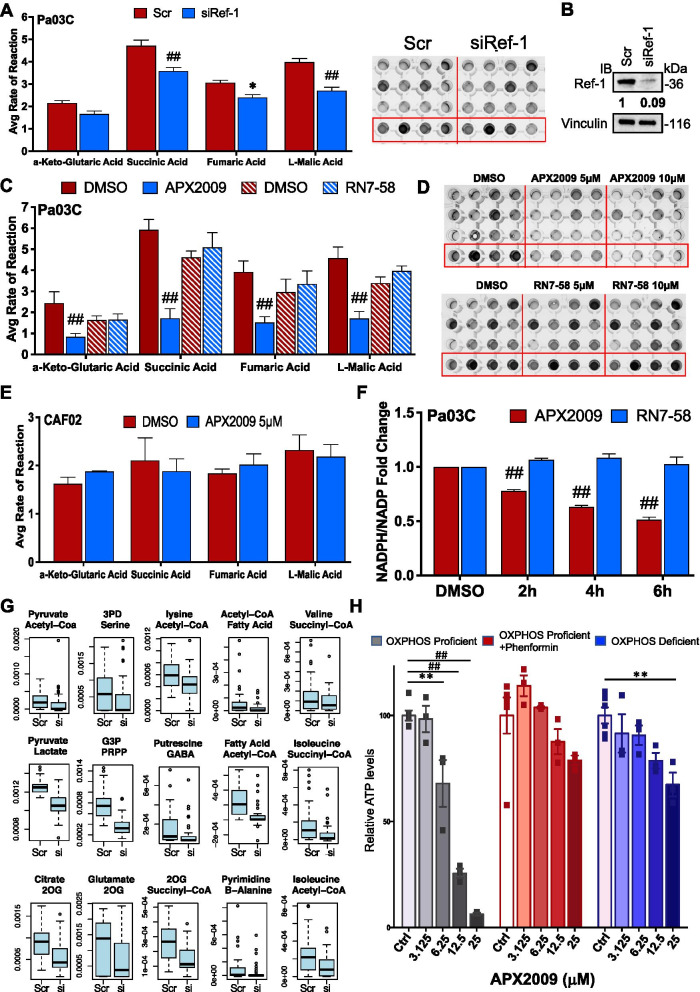
Ref-1 genetic or pharmacological inhibition reduces TCA cycle substrates. Mitochondrial functional assays in Pa03C cells transfected with Scr vs 10 nM siRef-1 (**A***n* = 3, *p < 0.05, ^##^0.0001) and a representative image of the plate. Avg rate of reaction refers to slope of absorbance at 590 vs time. Western blot image of the Pa03C cells after transfection with Ref-1 or Scr siRNA (**B**). Vinculin is used as the loading control. Average rate of reaction in Pa03C cells treated with Ref-1 redox inhibitor (APX2009, *n* = 3, *p < 0.05, ^##^0.0001) or inactive Ref-1 redox inhibitor analog (RN7-58, *n* = 2) (**C**) and their representative plate images (**D**) for 24 h. **E** Average rate of reaction in CAF02 cells treated with 5 µM APX2009 for 24 h (*n* = 3). **F** Fold change of the ratio of NADPH/NADP + in Pa03C cells treated with APX2009 (20 µM) or RN7-58 (20 µM) (*n* > 2, ^##^*p* < 0.0001). **G** Boxplots show estimated flux of NADP + consuming reactions in one metabolic module using scRNA seq data from Scr vs siRef-1. The two metabolite names on top of each plot are the input and output of each metabolic module. **H** Measurement of ATP levels by CellTiter-Glo Luminescent Cell Viability Assay in OXPHOS proficient cells (143B WT) or treated with 300 μM phenformin and OXPHOS deficient cells (143B CytB) after treatment with Ref-1 inhibitor APX2009 at the indicated concentrations for 24 h (**p < 0.01, ^##^0.0001). All data represent Mean ± SE

To confirm the gene expression and proteomic effects on TCA cycle and also investigate effects on fatty acid oxidation with functional data, a plate-based mitochondrial function assay that measures the electron flow through the electron transport chain was conducted (Supplemental Fig. S3A). This colorimetric assay measured the utilization rate of different metabolic substrates following knockdown of Ref-1 compared to Scr control. Multiple substrates involved in the TCA cycle exhibited significantly reduced rates of reaction after transfection with 10 nM siRef-1 (efficiency of Ref-1 knockdown (99%) shown in Fig. 4B) in comparison to 10 nM Scr siRNA (Fig. 4A). Succinic Acid was reduced by 24%, Fumaric Acid reduced by 22%, and Malic Acid was down by 33%. Changes in fatty acid oxidation was not observed under these conditons with these mitochondrial plates (not shown). The kinetic curves at 37ºC for the four TCA cycle substrates within a representative experiment are shown in Supplemental Figure S3B.

### Functional validation of the defect in metabolic pathways following pharmacological inhibition of Ref-1 redox activity and metabolic inhibitor, Devimistat

In order to determine whether Ref-1 redox activity was responsible for the decrease in TCA substrate activity, mitochondrial function assays were performed following APX2009 treatment at 5 and 10 µM. The four TCA cycle substrates (α-Keto-Glutaric Acid, Succinic Acid, Fumaric Acid, and Malic Acid, Supplemental Fig. S3A, C) displayed significantly reduced rates of reaction (30–72%) in response to 5 µM APX2009 treatment, and further dose-dependent reduction was observed when treated with 10 µM APX2009 (Supplemental Fig. S3C). These results confirm that Ref-1 redox activity plays a role in tumor cells’ ability to utilize TCA cycle substrates (Fig. 4C, D). As a negative control for this redox inhibition, an inactive analog of APX2009, RN7-58, was evaluated for its ability to block mitochondrial function, and no inhibition of TCA substrates was observed (Fig. 4C, D and Supplemental Fig. S3D) [67]. We also used a metabolic inhibitor, Devimistat as a positive control to compare and validate the effects on the TCA cycle by APX2009. Devimistat was able to significantly reduce two of the TCA cycle substrates (Malic acid by 20–30% and Fumaric Acid by 35% at 50 µM, Supplemental Fig. S3E and F). Similar to changes in mitochondrial complex gene expression in CAFs, APX2009 did not affect mitochondrial function within CAF02 cells (Fig. 4E and Supplemental Fig. S3G). Line graphs showing kinetic curves at 37ºC for the four TCA cycle substrates are represented by Supplemental Figure S3B-D, F and G.

Many of the genes that are affected by Ref-1 inhibition under hypoxia are likely to impact NADPH levels e.g. NNT, IDH2. Furthermore, NADPH scavenges reactive oxygen species and thus maintains redox homeostasis within cells. We measured NADPH/NADP ratios following treatment with APX2009 in Fig. 4F. Pa03C cells treated with APX2009 displayed significantly reduced levels of NADPH as observed from the NADPH/NADP ratio signifying the role of Ref-1 in sustaining cancer permissive environment. Importantly, the cells treated with Ref-1 redox inactive analog RN7-58 did not demonstrate any effect on the redox homeostasis of the cell (Fig. 4F).

To evaluate the effect of siRef-1 on NADP + /NADPH ratio, we also applied our in-house method single-cell flux estimation analysis (scFEA) to the hypoxia data [68]. scFEA utilizes a graph neural network model to estimate cell-wise metabolic flux by using scRNA-seq data. We specifically evaluated the NADP + consuming reactions in 15 metabolic modules (Fig. 4G). We observed the Scr cells have a higher flux rate of NADP + consuming reactions in all the 15 modules compared to siRef-1 cells. Our results suggested the less availability of NADP + in siRef-1 cells (Supplemental Fig. S3H), i.e., these cells may potentially bear a higher level of oxidative stress.

Based on the dramatic effect of Ref-1 on OXPHOS gene expression and TCA substrates, the effects of Ref-1 inhibition on OXPHOS deficient and proficient cells were evaluated [69]. Figure 4H shows that cells with functional OXPHOS are sensitive to Ref-1 inhibition as expected, however in contrast the OXPHOS deficient cells no longer respond to Ref-1 inhibition. Similarly, OXPHOS proficient cells treated with complex I inhibitor, Phenformin to mimic OXPHOS deficiency are also no longer sensitive to APX2009. This strengthens our hypothesis that inhibition of Ref-1 redox activity via APX2009 is mediating some of its cellular effects through a blockade of OXPHOS.

### Ref-1 redox inhibitor APX2009 and metabolic inhibitor Devimistat exhibit similar tumor reduction in in vivo xenograft studies

**Fig. 5:**
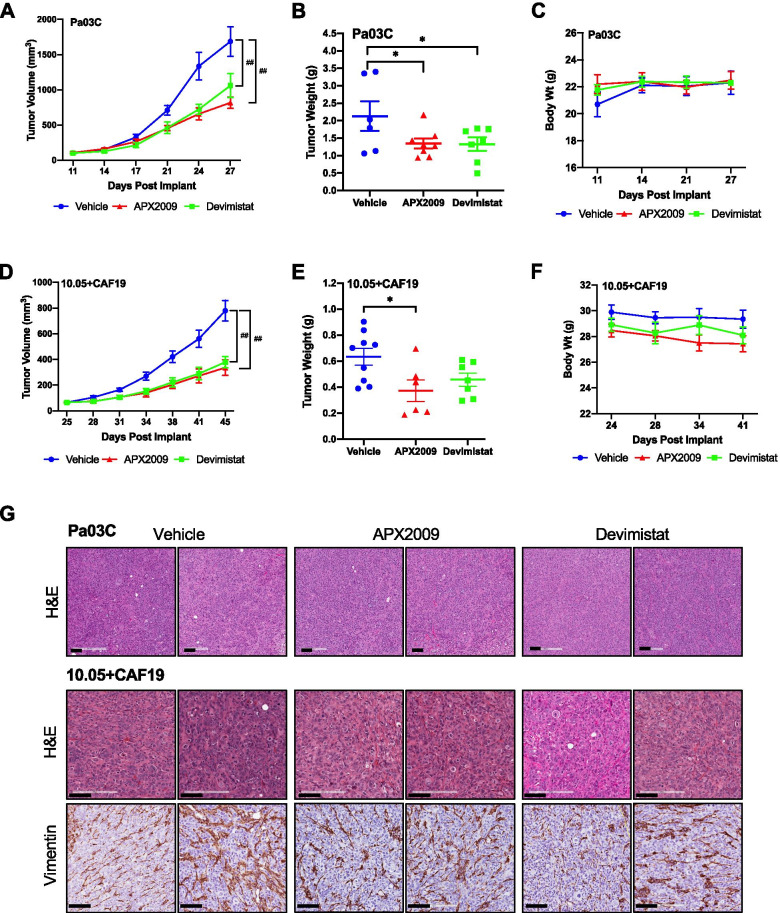
Ref-1 inhibition with APX2009 results in diminished tumor growth. Tumor growth of Pa03C (**A**) or Panc10.05 + CAF19 (**D**) subcutaneous xenografts treated with PKT Vehicle or 35 mg/Kg APX2009 or 50 mg/Kg Devimistat (^##^*p* < 0.0001, compared to vehicle control) twice a day, 8 h apart, continuously for 15 (for Pa03C xenografts) or 20 (for Panc10.05 + CAF19 xenografts) days. And corresponding tumor weights (**B**, **E** *p < 0.05, compared to vehicle control). **C**&**F** Graphs representing body weights over time for Pa03C or Panc10.05 + CAF19 xenografts. Representative images for H&E and IHC staining for vimentin positivity for Pa03C tumors and Panc10.05 + CAF19 (**G**). All data are represented as Mean ± SE

To determine whether the inhibition of Ref-1 leading to a reduction in mitochondrial gene expression and TCA substrate utilization would translate to a blockade of tumor growth in vivo, Pa03C cells were implanted in mice and treated with either APX2009 (35 mg/Kg) or metabolic inhibitor, Devimistat (50 mg/Kg). Both treatments resulted in a significant decrease in tumor volume over time starting at Day 17 (*p* < 0.0001, Fig. 5A). Growth rate per day of tumor volume among the groups was determined to be significantly different using the repeated measure regression model (*p* < 0.0001). Tumor weights also showed a significant decrease compared to the vehicle control group (*p* < 0.05) (Fig. 5B), with APX2009 and Devimistat showing a ~ 57% reduction in tumor weight. Both treatments were well tolerated as there was no significant change in body weights (Fig. 5C).

Due to the importance of the CAFs in the microenvironment and their impact on response to therapy, a tumor model with co-implantation of low passage pancreatic cancer cells, Panc10.05 and CAF19 cells was utilized. Similar to the results in Fig. 5A, treatment with either APX2009 (35 mg/Kg) or Devimistat (50 mg/Kg) resulted in a significant decrease in tumor volume (*p* < 0.0001, Fig. 5D). At the time of harvest, while both treatments showed a decrease in tumor weight, only APX2009 resulted in a statistically significant decrease by ~ 61% (*p* < 0.05, Fig. 5E). No significant change in body weights was observed (Fig. 5F). Tumors were collected and stained for H&E and Vimentin. Vimentin staining shows the presence of CAFs even after tumor shrinkage with APX2009 treatment (Fig. 5G and Supplemental Fig. S4A). Staining of the tumors wth hypoxia marker, CA9 demonstrates the presence of hypoxia in these in vivo models and underscores the importance of hypoxia signaling in the tumors’ response (Supplemental Fig. S4B).

### Inhibition of Ref-1 redox activity in combination with metabolic inhibitor Devimistat significantly reduces tumor growth in two 3D models with simulated PDAC microenvironment

**Fig. 6:**
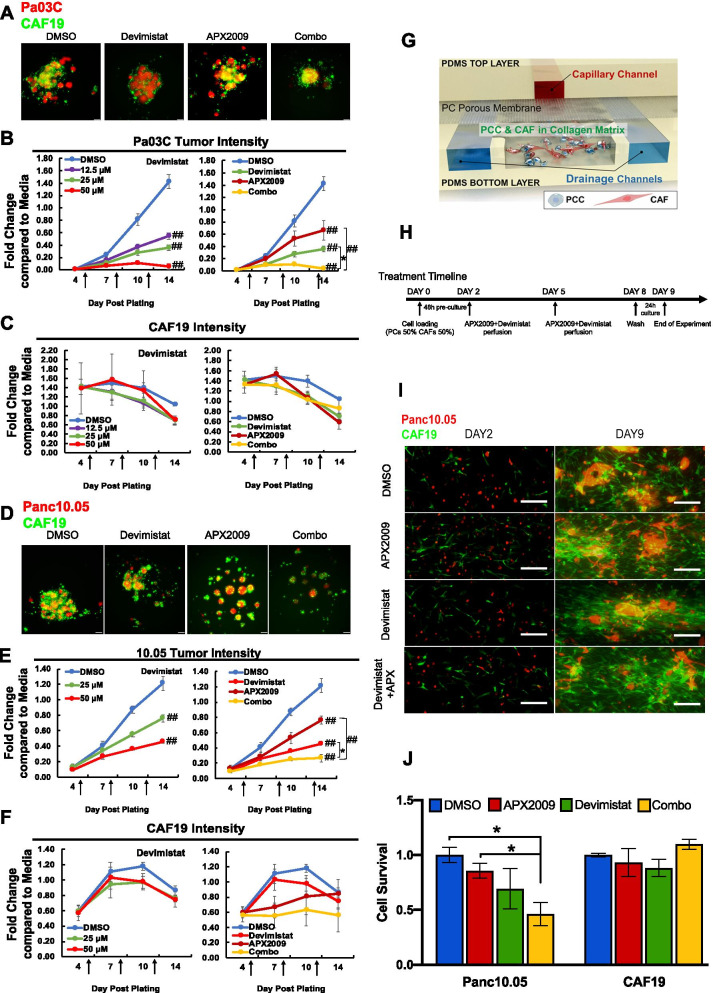
Ref-1 inhibition in combination with Devimistat attenuates growth in two co-culture models of pancreatic cancer: 3D spheroids and i-TMOC. Representative pictures of two low passage patient-derived low passage cell lines, Pa03C (**A**) and Panc10.05 (**D**) plated as 3D co-cultures with CAF19 cells at a ratio of 1:4. These co-cultures were treated with increasing concentrations of Devimistat (0–50 µM) and in combination with APX2009 following intensity measurements on Days 4, 7, 10, and 14. For combination treatment in Pa03C cells, Devimistat was held constant at 25 µM and APX2009 at 5 µM, and in Panc10.05, Devimistat was held constant at 50 µM and APX2009 at 10 µM (*p < 0.05, ^##^0.0001). Intensity of the tumor cells (red (**B**,**E**)) as well as the CAFs (green (**C**,**F**)) are represented as fluorescence intensity data normalized to Day 14 media control. Graphs are means with standard error of *n* = 3–4 and arrows correspond to treatment times. **G** Schematic of functional structure of PDAC iT-MOC and experimental timeline in (**H**). **I** Fluorescent microscopic observation of Panc10.05 (red) and CAF19 (green) in PDAC iT-MOC on Day 2 and Day 9. **J** Quantitation of cell survival in the iT-MOC system with single agent (APX2009 – 30 µM / Devimistat – 25 µM) and combination treatment (*n* ≥ 3, Mean ± S.E. *p < 0.05)

To assess whether the combination of APX2009 and Devimistat would synergize to prevent further tumor growth, two 3D co-culture assays were utilized. These included a 3D co-culture spheroid assay and an interstitial tumor-microenvironment-on-a-chip (iT-MOC) assay. Our group previously published doses of APX compounds that block spheroid growth in the 3D co-culture model [53]. Hence, the dose response of Devimistat as a single agent is shown in Pa03C (Fig. 6B, left panel) and in Panc10.05 cells (Fig. 6E, left panel). APX2009, parent drug APX3330 as well as additional analog APX2014, were used to inhibit Ref-1 redox activity as single agent and in combination with Devimistat (Fig. 6B, C, E, F and Supplemental Figure S5A). Single agent Devimistat or APX2009 resulted in significant reduction in spheroid growth as visualized through fluorescence intensity (Fig. 6A-F). However, combination treatment of Ref-1 inhibitor plus Devimistat resulted in further reduction of spheroid growth of the tumor cells (Fig. 6B & E). APX2009 + Devimistat had combination index (CI) values ranging from 0.37–0.74 indicating moderate to strong synergy (see Supplementary Table 6 for all values). Importantly, the single agent and combination treatments had little to no effect on CAF19 growth (Fig. 6A-F, with the exception of combination treatment in the Panc10.05 + CAF19 co-cultures (Fig. 6F, right panel, Supplemental Fig. S5A). Supplemental Figure S5A shows similar results with the Ref-1 redox inhibitors, APX3330 (completed Phase I clinical trial) and APX2014 in combination with Devimistat.

Combination drug experiments were also performed using the iT-MOC assays with the co-culture of Panc10.05 and CAF19 cells [70]. This assay was utilized due to its ability to reconstitute the interstitial transport in a 3D matrix (Fig. 6G). The capillary channel mimics blood-borne drug transport along a capillary vessel. The porous membrane simulates transvascular transport. The interstitial channel where pancreatic cancer cells and CAFs are co-cultured in the 3D matrix aims to reconstitute the interstitial transport. Two side drainage channels correspond to lymphatic drainage. The drug transport is achieved by applying a hydrostatic pressure difference between the capillary channel and lymphatic channel, simulating elevated interstitial fluid pressure (IFP) range in PDAC. Transport properties of iT-MOC have been measured and compared with in vivo tumors previously [57, 58].

Figure 6H represents the treatment timeline for iT-MOC assay. Representative fluorescence micrographs of iT-MOC samples during the experiment are shown in Fig. 6I. In the control group, both cell types proliferated significantly over time as indicated by red and green fluorescence (Fig. 6I, Supplemental Fig. S5B). In terms of cell growth (Supplemental Fig. S5B) and survival (Fig. 6J), no notable difference is observed in cancer cell growth among the experimental groups until Day 5. After the second treatment at Day 5, tumor cells treated with a combination of APX2009 and Devimistat grew much slower at 48% of the control group compared to either single agent (*p* < 0.05, Fig. 6J & Supplemental Fig. S5B). In all experimental groups, no notable difference in the proliferation of CAFs is noted (Fig. 6J & Supplemental Fig. S5C) indicating that the combination treatment was more dramatically affecting the tumor even in the presence of the CAFs and was similar in both 3D co-culture assays.

### Inhibition of Ref-1 redox activity in combination with metabolic inhibitor Devimistat reduces mitochondrial gene expression and causes TCA cycle disruption

**Fig. 7:**
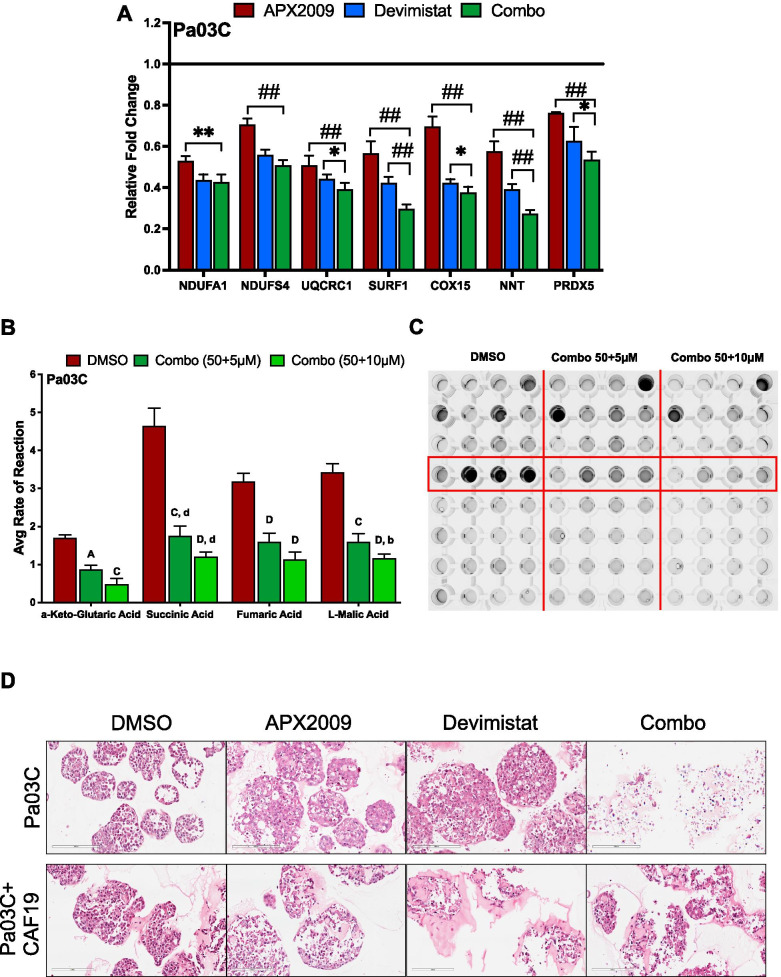
Ref-1 inhibition in combination with Devimistat downregulates gene expression of the mitochondrial complex genes as well as mitochondrial function. Expression of mitochondrial genes (**A**) via qPCR in Pa03C 3D spheroids treated with DMSO or a combination of APX2009 (5 µM) and Devimistat (50 µM) (*n* = 3, *p* < 0.05–0.0001). The data with APX2009 is also in Fig. 3I and provided here for comparison to combination. **B** Mitochondrial functional assay in Pa03C with DMSO or a combination of APX2009 (5 & 10 µM, 24 h) and Deviminstat (50 µM, 24 h) (*n* = 3, *p* < 0.01, 0.0001) and the representative image of the plate following the reaction (**C**). Data represent Mean ± SE; Uppercase letters denote statistical significance of combination treatment to APX2009 (5 µM) and lowercase letters denote statistical significance of combination treatment to Devimistat (50 µM). **D** H&E staining of spheroids from Pa03C alone or cultures with CAF19 cells treated with APX2009 (5 µM) or Devimistat (50 µM) or a combination in comparison to DMSO control

Based on the 3D data demonstrating efficacy of the combination treatment, we further investigated the effects on gene expression of combination treatment in Pa03C spheroids. Of the six previously tested OXPHOS mitochondrial genes found from the scRNA-seq data, the combination treatment (blue bars) significantly reduced the levels of UQCRC1, SURF1, COX15 and NNT compared to either of the single agents used (Fig. 7A). Within the Ref-1-regulated 12 gene panel, combination treatment reduced the levels of NOTCH3 (which was not affected by either single agent) and RAB3D while increasing the levels of PPIF significantly compared to either singles. The effects of combination treatment on ABCG2, CIRBP, COMMD7, ISYNA1, PRDX5 and TNFAIP2 were significantly down compared to either APX2009 alone or Devimistat alone, however the combination effects were most dramatic in NOTCH3 and RAB3D. BIRC5 and CA9 were used as controls (Supplemental Figure S6A).

Following this, we analyzed whether the reduced gene expression with combination treatment had a further functional effect on the TCA cycle. For this, a combination of Devimistat (50 µM) with APX2009 (5 or 10 µM) was used. Devimistat with APX2009 at 10 µM had a greater effect on reducing the rate of reaction of all four TCA substrates compared to APX2009 or Devimistat alone (α-Keto-Glutaric Acid – 42 or 58%, Succinic Acid – 30 or 64%, Fumaric Acid – 25 or 12.5%, and Malic Acid – 32 or 46%) (Fig. 7B). Representative picture of the reduction in colorimetric assay shown in Fig. 7C. The effects were also dose-dependent. As the amount of APX2009 increased, the TCA substrate utilization was decreased. Line graphs showing kinetic curves (Supplemental Fig. S6B) at 37ºC for the four TCA cycle substrates are represented.

3D spheroids of Pa03C alone or co-cultured with CAF19 cells were stained with H&E to visualize the effects of single agent treatment with Ref-1 redox inhibition, APX2009 or metabolic inhibition, Devimistat, or the combination. Staining of the 3D spheroids following combination treatment dramatically demonstrated the anti-proliferative effect of the combination treatment in Fig. 7D. Moreover, the stroma again appears less affected, similar to the in vivo experiment with the single agents.

## Discussion

A hallmark of PDAC is its capability to reprogram metabolism. Pancreatic cancer’s ability to adapt to nutrient and oxygen fluctuation results in severe therapeutic resistance. Communicating with its tumor microenvironment (TME) through reciprocal upregulation or downregulation of several metabolic pathways like aerobic glycolysis, oxidative phosphorylation, glutaminolysis, lipogenesis and lipolysis, autophagic status, and anti-oxidative stress enables PDAC to thrive under nutrient deficiency and low oxygen conditions, as well as evade therapeutic death. KRAS oncogenic driver mutations are responsible for these tumor cells highly relying on OXPHOS for survival (by supplying ATP) as well as drug resistance (via multidrug transporters) [71, 72]. Current options for the treatment of PDAC patients are FOLFIRINOX and gemcitabine + nab-paclitaxel. However these multi-agent options are still not adequate to cure pancreatic cancer and are often toxic to the patients [73]. Hence, this study investigates the targeting of metabolic pathways through Ref-1 inhibition as an option for therapy.

Our scRNA-seq analysis of Pa03C cells transfected with Scr or siRef-1 under hypoxia identified novel pathways and transcriptional modules regulated by Ref-1, thereby opening up new avenues to treat PDAC. As represented in the Fig. 1D panel 2, pathway enrichment analysis revealed TCA cycle, lipid metabolism, glycolysis, OXPHOS and HIF1α pathways as the top five to be downregulated with Ref-1 knockdown under hypoxia. Further detailed analysis combining scRNA-seq, proteomics, and functional assays revealed the enzymes involved in those selected pathways to be clustered to TCA cycle and OXPHOS (Fig. 2). With the intent of validating these effects, we selected a set of genes representive of the above pathways and observed a significant reduction in their levels with Ref-1 knockdown (Fig. 3A-C and Supplemental Fig. S1A-D) as well as Ref-1 redox signaling inhibition with APX2009 (Fig. 3E–G and Supplemental Fig. S1H-M). We also noted similar reduction at protein level with Ref-1 knockdown (Fig. 3D and Supplemental Fig. S1E-G) as represented by ACLY, IDH2 and SURF1 proteins under both normoxia and hypoxia. Furthermore, we observed a decrease in the NADPH/NADP ratio indicating that there was a more oxidized environment when Ref-1 redox activity was blocked (Fig. 4F). Metabolic pathways generate energy for cancer survival and progression in the form of NADH / NADPH / FADH and ATP. NADPH is the reduced high energy molecule that can be readily used by the cancer cells for anabolic reactions. Ref-1 inhibition with APX2009 causes a decrease in NADPH levels confirming the predicted higher flux rate of NADP + consuming reactions resulting in a higher level of oxidative stress observed in Ref-1 knockdown (Fig. 4G). Similar to previous studies surveying pathways affected by Ref-1 silencing in cancer cells, mitochondrial function was found as one of the most significantly changed, including our previous scRNA seq study in PDAC cells [25, 74, 75]. What was not clear from these studies was whether the DNA BER function or the redox signaling function of Ref-1 was responsible for this perturbation of mitochondrial function. The results presented here demonstrate that a second generation, more potent Ref-1 redox inhibitor, APX2009, significantly downregulated all OXPHOS-associated genes (Fig. 3E&F). This points toward the transcriptional regulation of OXPHOS through Ref-1 redox signaling as the main determinant of the effects on mitochondrial function when Ref-1 is knocked down. We further verified this using the OXPHOS deficient cells in Fig. 4H. Our hypothesis that Ref-1 inhibition resulted in a lack of cell viability through the blockade of OXPHOS was corroborated by the data showing that OXPHOS deficient cells are no longer sensitive to Ref-1 inhibition. We will be utilizing this cell system to further understand the contribution Ref-1 redox signaling on OXPHOS and ways to exploit the cells’ dependence on metabolism for survival.

Another interesting gene expression change is the upregulation of PPIF (peptidylprolyl isomerase F or cyclophilin D). PPIF is part of the mitochondrial permeability transition pore (PTP) that is invoved in the induction of cell death [76]. Interestingly, under hypoxia PPIF is significantly downregulated, presumably one of the many transcriptional changes that allow cancer cells to survive hypoxic stress. However, with APX2009 or Devimistat treatment or combination of the two, the levels of PPIF are increased about 5–tenfold (Supplemental Fig. S6A) indicating that inhibition of TCA is likely increasing tumor cell death. The inhibition of TCA cycle activity was confirmed with APX2009, Devimistat, or combination therapy using a functional assay. Either agent used alone affected the TCA substrates with APX2009 being more potent than Devimistat. These data indicate that Ref-1 redox signaling inhibition reduces the activity of the TCA cycle similar to Devimistat, but at a tenfold lower concentration, leading to cell death (Fig. 7B).

CAFs, which are most abundant in the TME, form a critical component of the PDAC milieu. CAFs produce lactate from glycolysis that fuels OXPHOS in the PDAC cells for ATP production via alpha-ketoglutarate, isocitrate dehydrogenase and pyruvate dehydrogenase enzymes. The production of L-lactate, ketones, free fatty acids, and glutamine by CAFs may also drive tumor cell growth through metabolic coupling and enabling the tumor to utilize OXPHOS. HIF1α, NFκB, and loss of Cav-1 have been implicated in the signaling that drives autophagy and catabolism in CAFs that can directly affect tumor growth. Thus, tumors switch between OXPHOS and glycolysis under diverse microenvironmental conditions [77]. Both transcription factors, HIF1α, and NFκB are targets of Ref-1 redox signaling activity [78–80]. Hence, targeting both OXPHOS in cancer cells as well as glycolysis in CAFs may prove effective, with the caveat that toxicity to non-cancerous cells must be considered [79, 81]. Ref-1 redox inhibition showed minimal effects on the expression of mitochondria complex genes and on mitochondria function in the CAFs (Figs. 3H and 4E). Noticeably, the effects of Ref-1 inhibition on mitochondrial function is not the same between tumor and CAFs, pointing again toward a different mechanism of metabolic reprogramming regarding tumor and CAF cells. Furthermore, the effects of Devimistat as a single agent or in combination therapy in the 3D models shown in Fig. 6 demonstrate that targeting of the TCA cycle is more cytotoxic to the tumor cells than the CAFs. The 3D co-culture spheroids consisting of both tumor cells and CAFs demonstrate regions of hypoxia and growth factor gradients [53]. 3D coculture assays demonstrated that the combination treatment was not only more effective than the single agents, but that the treatments in general targeted the tumor cells more dramatically than the surrounding microenvironment with minimal effects on the CAFs (Fig. 6A-F). In the iT-MOC system, fluorescence from both cell types significantly increased over time, while in the 3D spheroid based assay the CAFs are less proliferative than the tumors (Fig. 6 and Supplementary Fig. S5). Likewise, combination of APX2009 with Devimistat proved to significantly reduce the expression of all six mitochondrial complex genes when compared to single agents in 3D spheroids consisting of Pa03C cells (Fig. 7A). Devimistat is inhibiting PDH (pyruvate dehydrogenase) and αKGDH (α-ketoglutarate dehydrogenase) and thereby blocking TCA cycle activity, whereby inhibition of Ref-1 appears to dramatically affect the expression of several genes involved in various aspects of metabolism including glycolysis, TCA cycle, and ETC, with TCA cycle being the most downregulated pathway following knockdown in hypoxia (Fig. 1D). Future studies will further define the metabolic pathways that are utilized during varying oxygen levels in the presence and absence of Ref-1 and whether there is a shift to TCA cycle at extreme oxygen deprivation as described in [26]. The ROS levels generated during hypoxia as well as Ref-1 knockdown are of interest, as well as activaton of NFκB [28]. Ostensibly, the combination therapy is further shutting down the tumor cells’ ability to generate energy and leading to a decrease in 3D spheroid tumor growth. Tumor-selective killing is desirable in PDAC therapeutic approaches, as attempts to modulate the PDAC stroma to improve treatment response have not been well translated and, in fact, have shown to hinder therapeutic approaches. Due to an unanticipated anti-tumor role of select stroma components, the ablation of CAFs or targeting of certain ECM proteins in the stroma led to changes that actually accelerated tumor growth and impaired treatment outcome [82–84].

To demonstrate that inhibition of Ref-1 redox activity blocks tumor growth in vivo and also assess the effects on the CAFs, low passage PDAC cells were implanted alongside CAFs into NSG mice. Treatment with either APX2009 or Devimistat which should lead to a decrease in cancer cell metabolism did indeed translate to a significant decrease in tumor volume (Fig. 5A & D) as well as tumor weight (Fig. 5B & E) and did not obliterate the CAFs as indicated by vimentin positivity. These in vivo xenograft results suggest that targeting the metabolic capacity of PDAC tumors will slow their growth rate without killing the associated stroma have strong translational implications.

Several of the transcription factors that interact with Ref-1 have a profound impact on metabolic reprogramming in cancer, including HIF1-α, STAT3, and NRF2 (nuclear factor erythroid 2-related factor 2) [85, 86]. NRF2 is a transcription factor that can act in a protective mechanism in cancer cells from oxidative and chemical stresses by controlling their redox balance, regulation of antioxidant genes, and metabolic reprogramming [86]. Interestingly, Ref-1 redox signaling activity activates HIF1-α and STAT3 while the opposite is true of NRF2. Conceivably by blocking Ref-1 redox activity, the tumor cells’ ability to switch to aerobic glycolysis is attenuated and thereby the tumor cells are less proliferative. Our previously published work demonstrated that, selective inactivation of Ref-1 leads to upregulation of NRF2 target genes like HMOX-1 [23]. This could be the cellular response to the blockade by Ref-1 on the TCA cycle. Taken together from our current work, it is clear that more research is needed to fully understand the intersection of Ref-1 signaling and transcriptional regulation of metabolism and its impact on cancer cell growth.

## Conclusion

This is the first study to identify Ref-1’s role in regulating glycolysis and mitochondrial metabolism through the integration of single cell RNA seq, proteomics, and mitochondrial function under hypoxia and Ref-1 deficient redox signaling conditions. Ref-1’s redox signaling / regulatory role plays a crucial function in mitochondrial metabolism, specifically altering the TCA cycle and gene expression within OXPHOS / ETC thereby enabling the survival of PDAC even under nutrient and oxygen stress. We used 3D spheroids in monoculture as a mechanistic model or as co-culture with CAFs as a means to demonstrate growth regulation by Ref-1 both in vitro as well as in vivo. In these models, we determined that Ref-1 inhibition leads to significant reduction in PDAC growth and in regulation of gene expression that leads to the dysregulation of many genes within Complex 1 of the ETC especially under hypoxia (Fig. 2C).

As mitochondria can also generate reactive oxygen species (ROS) that damage nuclear and mitochondrial DNA, the role of Ref-1’s BER function was hypothesized to also be important in the observed phenotype, as seen in the experiments with Ref-1 siRNA knockdown. Several studies including ours demonstrated that the repair role of Ref-1 (APE1) is essential to maintain the integrity of mitochondria after oxidative stress [24, 87, 88]. Ref-1’s redox regulation of transcription factors leading to gene expression changes that ultimately regulate mitochondrial function has been less explored. APX3330 and its analogs, APX2009 and others, specifically target Ref-1’s redox signaling activity, but not the DNA repair function [66]. Therefore, this study in addition to exploring the consequences of targeting mitochondrial dysfunction in PDAC, demonstrates that the redox activity of Ref-1 plays a novel role in metabolic signaling through disruption of the TCA cycle and the ETC. Although many studies have shown mitochondrial perturbation following Ref-1 blockade, we are the first to implicate the redox signaling function in this phenotype.

## Supplementary Information

**Additional file 1: Supplemental Methods. Supplementary Figure 1.** Interrogation of gene and protein expression following Ref-1 inhibition. **Supplementary Figure 2.** Ref-1 downregulates growth of 3D spheroids in low glucose containing media conditions. **Supplementary Figure 3.** Ref-1 genetic or pharmacological inhibition reduces TCA cycle substrates. **Supplementary Figure 4.** IHC markers (vimentin and CA9) on tumors after treatment with APX2009 or Devimistat. **Supplementary Figure 5.** Ref-1 inhibition with additional APX analogs in combination with Devimistat attenuates growth in two co-culture models of pancreatic cancer: 3D spheroids and i-TMOC. **Supplementary Figure 6.** Ref-1 inhibition in combination with Devimistat shifts metabolism significantly compared to single agents. **Supplemental Table 1.** TMT labels used for each sample. **Supplemental Table 2.** qPCR Primers used for qRT-PCR. **Supplemental Table 3.** Complete lists of the dysregulated genes and proteins. **Supplemental Table 4.** Complete lists of the differentially expressed pathways. **Supplemental Table 5.** Predicted transcriptional regulatory factors of each identified module. **Supplemental Table 6.** Combination index (CI) values for the 3D assay with APX2009 in combination with Devimistat.

**Additional file 2.**

**Additional file 3.**

**Additional file 4.**

**Additional file 5.**

## Data Availability

The previously published scRNA-seq data under normoxia can be found in the GEO, accession number GSE99305 and the hypoxia data presented here is also in GEO, accession number GSE173433. The mass spectrometry proteomics data have been deposited to the ProteomeXchange Consortium via the PRIDE [89] partner repository with the dataset identifier PXD020515 and 10.6019/PXD020515. The single cell hypoxia data is currently being submitted to the GEO database.

## Abbreviations

AP-1: Activator protein-1
BER: Base excision repair
CAFs: Cancer-associated fibroblasts
DEGs: Differentially expressed genes
DMSO: Dimethylsulfoxide
ECM: Extracellular matrix
ETC: Electron transport chain
HIF1-α: Hypoxia inducible factor 1- alpha
HMOX-1: Heme oxygenase 1
IFP: Interstitial fluid pressure
iT-MOC: Interstitial tumor-microenvironment-on-chip
NADPH/NADP: Nicotinamide adenine dinucleotide phosphate
NFkB: Nuclear factor kappa b
NSG: NOD scid gamma (NOD.Cg-*Prkdc*^*scid*^* Il2rg*^*tm1Wjl*^/SzJ)
NRF2: Nuclear factor erythroid 2-related factor 2
OXPHOS: Oxidative phosphorylation
PDAC: Pancreatic ductal adencarcinoma
PDH: Pyruvate dehydrogenase
PKT: Propylene Glycol, Kolliphor HS15, Tween 80
qRT-PCR: Quantitative real time-polymerase chain reaction
Ref-1/APE1: Redox factor 1/Apurinic-apyrimidinic endonuclease 1
ROS: Reactive oxygen species
Scr: Scrambled
scRNA-seq: Single cell RNA sequencing
STAT3: Signal transducer and activator of transcription 3
TCA cycle: Tricarboxylic acid cycle
TF: Transcription factors
TME: Tumor microenvironment

## References

[1] Siegel RL, Miller KD, Jemal A (2019). Cancer statistics, 2019. CA Cancer J Clin.

[2] Bijlsma MF, van Laarhoven HW (2015). The conflicting roles of tumor stroma in pancreatic cancer and their contribution to the failure of clinical trials: a systematic review and critical appraisal. Cancer Metastasis Rev.

[3] Longati P (2013). 3D pancreatic carcinoma spheroids induce a matrix-rich, chemoresistant phenotype offering a better model for drug testing. BMC Cancer.

[4] Chang Q, Jurisica I, Do T, Hedley DW (2011). Hypoxia predicts aggressive growth and spontaneous metastasis formation from orthotopically grown primary xenografts of human pancreatic cancer. Cancer Res.

[5] Rucki AA (2016). Heterogeneous stromal signaling within the tumor microenvironment controls the metastasis of pancreatic cancer. Cancer Res.

[6] Chaika NV (2012). Differential expression of metabolic genes in tumor and stromal components of primary and metastatic loci in pancreatic adenocarcinoma. PLoS One.

[7] Ngoi NYL (2020). Targeting cell metabolism as cancer therapy. Antioxid Redox Signal.

[8] Biancur DE, Kimmelman AC (2018). The plasticity of pancreatic cancer metabolism in tumor progression and therapeutic resistance. Biochim Biophys Acta Rev Cancer.

[9] Zhang J, Pavlova NN, Thompson CB (2017). Cancer cell metabolism: the essential role of the nonessential amino acid, glutamine. EMBO J.

[10] DeBerardinis RJ, Cheng T (2010). Q’s next: the diverse functions of glutamine in metabolism, cell biology and cancer. Oncogene.

[11] Smolkova K (2011). Waves of gene regulation suppress and then restore oxidative phosphorylation in cancer cells. Int J Biochem Cell Biol.

[12] Fan J (2013). Glutamine-driven oxidative phosphorylation is a major ATP source in transformed mammalian cells in both normoxia and hypoxia. Mol Syst Biol.

[13] Seagroves TN (2001). Transcription factor HIF-1 is a necessary mediator of the pasteur effect in mammalian cells. Mol Cell Biol.

[14] Robin ED, Murphy BJ, Theodore J (1984). Coordinate regulation of glycolysis by hypoxia in mammalian cells. J Cell Physiol.

[15] Fung H, Demple B (2005). A vital role for Ape1/Ref1 protein in repairing spontaneous DNA damage in human cells. Mol Cell.

[16] Izumi T (2005). Two essential but distinct functions of the mammalian abasic endonuclease. Proc Natl Acad Sci U S A.

[17] Jiang Y (2009). Role of APE1 in differentiated neuroblastoma SH-SY5Y cells in response to oxidative stress: use of APE1 small molecule inhibitors to delineate APE1 functions. DNA Repair (Amst).

[18] Kelley MR, Logsdon D, Fishel ML (2014). Targeting DNA repair pathways for cancer treatment: what’s new?. Future Oncol.

[19] Gaiddon C, Moorthy NC, Prives C (1999). Ref-1 regulates the transactivation and pro-apoptotic functions of p53 in vivo. EMBO J.

[20] Lando D, Pongratz I, Poellinger L, Whitelaw ML (2000). A redox mechanism controls differential DNA binding activities of hypoxia-inducible factor (HIF) 1alpha and the HIF-like factor. J Biol Chem.

[21] Cardoso AA (2012). APE1/Ref-1 regulates STAT3 transcriptional activity and APE1/Ref-1-STAT3 dual-targeting effectively inhibits pancreatic cancer cell survival. PLoS One.

[22] Kelley MR, Georgiadis MM, Fishel ML (2012). APE1/Ref-1role in redox signaling: translational applications of targeting the redox function of the DNA repair/redox protein APE1/Ref-1. Curr Mol Pharmacol.

[23] Fishel ML (2015). Apurinic/apyrimidinic endonuclease/redox factor-1 (APE1/Ref-1) redox function negatively regulates NRF2. J Biol Chem.

[24] Shah F, et al. Exploiting the Ref-1-APE1 node in cancer signaling and other diseases: from bench to clinic. NPJ Precis Oncol. 2017;1. 10.1038/s41698-017-0023-0. 10.1038/s41698-017-0023-0 PMC5558897 28825044

[25] Shah F (2017). APE1/Ref-1 knockdown in pancreatic ductal adenocarcinoma - characterizing gene expression changes and identifying novel pathways using single-cell RNA sequencing. Mol Oncol.

[26] Eales KL, Hollinshead KE, Tennant DA (2016). Hypoxia and metabolic adaptation of cancer cells. Oncogenesis.

[27] Vaziri-Gohar A, Zarei M, Brody JR, Winter JM (2018). Metabolic dependencies in pancreatic cancer. Front Oncol.

[28] Tang K (2019). Hypoxia-reprogrammed tricarboxylic acid cycle promotes the growth of human breast tumorigenic cells. Oncogene.

[29] Sharbeen G, McCarroll J, Goldstein D, Phillips P. Exploiting base excision repair to improve therapeutic approaches for pancreatic cancer. Front Nutr. 2015;2. 10.3389/fnut.2015.00010. 10.3389/fnut.2015.00010 PMC4428371 25988138

[30] Caston RA (2020). The multifunctional APE1 DNA repair–redox signaling protein as a drug target in human disease. Drug Discovery Today.

[31] Fishel ML (2011). Impact of APE1/Ref-1 redox inhibition on pancreatic tumor growth. Mol Cancer Ther.

[32] Jiang Y, Zhou S, Sandusky GE, Kelley MR, Fishel ML (2010). Reduced expression of DNA repair and redox signaling protein APE1/Ref-1 impairs human pancreatic cancer cell survival, proliferation, and cell cycle progression. Cancer Invest.

[33] Kelley MR (2016). Identification and characterization of new chemical entities targeting apurinic/apyrimidinic endonuclease 1 for the prevention of chemotherapy-induced peripheral neuropathy. J Pharmacol Exp Ther.

[34] McIlwain DW, Fishel ML, Boos A, Kelley MR, Jerde TJ (2018). APE1/Ref-1 redox-specific inhibition decreases survivin protein levels and induces cell cycle arrest in prostate cancer cells. Oncotarget.

[35] Sardar Pasha SPB (2018). Ref-1/APE1 inhibition with novel small molecules blocks ocular neovascularization. J Pharmacol Exp Ther.

[36] Fishel ML (2019). Antitumor activity and mechanistic characterization of APE1/Ref-1 inhibitors in bladder cancer. Mol Cancer Ther.

[37] Alistar A (2017). Safety and tolerability of the first-in-class agent CPI-613 in combination with modified FOLFIRINOX in patients with metastatic pancreatic cancer: a single-centre, open-label, dose-escalation, phase 1 trial. Lancet Oncol.

[38] Lee KC (2014). Translational assessment of mitochondrial dysfunction of pancreatic cancer from in vitro gene microarray and animal efficacy studies, to early clinical studies, via the novel tumor-specific anti-mitochondrial agent, CPI-613. Ann Transl Med.

[39] Stuart SD (2014). A strategically designed small molecule attacks alpha-ketoglutarate dehydrogenase in tumor cells through a redox process. Cancer Metab.

[40] Jones S (2008). Core signaling pathways in human pancreatic cancers revealed by global genomic analyses. Science.

[41] Logsdon DP (2016). Regulation of HIF1alpha under hypoxia by APE1/Ref-1 impacts CA9 expression: dual targeting in patient-derived 3D pancreatic cancer models. Mol Cancer Ther.

[42] Richards KE (2016). Cancer-associated fibroblast exosomes regulate survival and proliferation of pancreatic cancer cells. Oncogene.

[43] Principe DR (2020). Long-term gemcitabine treatment reshapes the pancreatic tumor microenvironment and sensitizes murine carcinoma to combination immunotherapy. Cancer Res.

[44] Wan C (2019). LTMG: a novel statistical modeling of transcriptional expression states in single-cell RNA-Seq data. Nucleic Acids Res.

[45] Wan C, et al. Fast and Efficient Boolean Matrix Factorization by Geometric Segmentation. Proceedings of the AAAI Conference on Artificial Intelligence. 2019. p. 6086–6093.

[46] Subramanian A (2005). Gene set enrichment analysis: a knowledge-based approach for interpreting genome-wide expression profiles. Proc Natl Acad Sci U S A.

[47] Peck Justice SA (2020). Mutant thermal proteome profiling for characterization of missense protein variants and their associated phenotypes within the proteome. J Biol Chem.

[48] Levasseur EM, et al. Hypusine biosynthesis in beta cells links polyamine metabolism to facultative cellular proliferation to maintain glucose homeostasis. Sci Signal. 2019;12. 10.1126/scisignal.aax0715. 10.1126/scisignal.aax0715 PMC7202401 31796630

[49] Fan Z (2003). Cleaving the oxidative repair protein Ape1 enhances cell death mediated by granzyme A. Nat Immunol.

[50] Wang D, Luo M, Kelley MR (2004). Human apurinic endonuclease 1 (APE1) expression and prognostic significance in osteosarcoma: enhanced sensitivity of osteosarcoma to DNA damaging agents using silencing RNA APE1 expression inhibition. Mol Cancer Ther.

[51] Fishel ML (2008). Knockdown of the DNA repair and redox signaling protein Ape1/Ref-1 blocks ovarian cancer cell and tumor growth. DNA Repair (Amst).

[52] Fishel ML, Colvin ES, Luo M, Kelley MR, Robertson KA (2010). Inhibition of the redox function of APE1/Ref-1 in myeloid leukemia cell lines results in a hypersensitive response to retinoic acid-induced differentiation and apoptosis. Exp Hematol.

[53] Logsdon DP (2018). Blocking HIF signaling via novel inhibitors of CA9 and APE1/Ref-1 dramatically affects pancreatic cancer cell survival. Sci Rep.

[54] Sempere LF, Gunn JR, Korc M (2011). A novel 3-dimensional culture system uncovers growth stimulatory actions by TGFbeta in pancreatic cancer cells. Cancer Biol Ther.

[55] Arpin CC (2016). Applying small molecule signal transducer and activator of transcription-3 (STAT3) protein inhibitors as pancreatic cancer therapeutics. Mol Cancer Ther.

[56] Lindblom P (2012). Tesaglitazar, a dual PPAR-alpha/gamma agonist, hamster carcinogenicity, investigative animal and clinical studies. Toxicol Pathol.

[57] Ozcelikkale A (2017). Differential response to doxorubicin in breast cancer subtypes simulated by a microfluidic tumor model. J Control Release.

[58] Kwak B, Ozcelikkale A, Shin CS, Park K, Han B (2014). Simulation of complex transport of nanoparticles around a tumor using tumor-microenvironment-on-chip. J Control Release.

[59] Shin CS, Kwak B, Han B, Park K (2013). Development of an in vitro 3D tumor model to study therapeutic efficiency of an anticancer drug. Mol Pharm.

[60] Fitzmaurice GM, Laird NM, Ware JH. Statistic in Medicine. In; Applied longitudinal analysis. Hoboken: Wiley Online Library; 2004.

[61] Baran-Gale J, Chandra T, Kirschner K (2018). Experimental design for single-cell RNA sequencing. Brief Funct Genomics.

[62] Ziegenhain C (2017). Comparative analysis of single-cell RNA sequencing methods. Mol Cell.

[63] Zhang Y (2019). M3S: a comprehensive model selection for multi-modal single-cell RNA sequencing data. BMC Bioinformatics.

[64] Xie J (2020). QUBIC2: a novel and robust biclustering algorithm for analyses and interpretation of large-scale RNA-Seq data. Bioinformatics.

[65] Wan C (2019). Association for the advancement of artificial intelligence.

[66] Luo M (2008). Role of the multifunctional DNA repair and redox signaling protein Ape1/Ref-1 in cancer and endothelial cells: small-molecule inhibition of the redox function of Ape1. Antioxid Redox Signal.

[67] Kelley MR (2011). Functional analysis of novel analogs of E3330 that block the redox signaling activity of the multifunctional AP endonuclease/redox signaling enzyme APE1/Ref-1. Antioxid Redox Signal.

[68] Alghamdi N, et al. A graph neural network model to estimate cell-wise metabolic flux using single cell RNA-seq data. bioRxiv. 2021;2020.2009.2023.310656. 10.1101/2020.09.23.310656. 10.1101/gr.271205.120 PMC8494226 34301623

[69] Madala HR (2020). Nitrogen trapping as a therapeutic strategy in tumors with mitochondrial dysfunction. Cancer Res.

[70] Moon HR (2020). An engineered pancreatic cancer model with intra-tumoral heterogeneity of driver mutations. Lab Chip.

[71] Zhou Y (2012). Intracellular ATP levels are a pivotal determinant of chemoresistance in colon cancer cells. Cancer Res.

[72] Viale A (2014). Oncogene ablation-resistant pancreatic cancer cells depend on mitochondrial function. Nature.

[73] Alistar A (2017). Safety and tolerability of the first-in-class agent CPI-613 in combination with modified FOLFIRINOX in patients with metastatic pancreatic cancer: a single-centre, open-label, dose-escalation, phase 1 trial. Lancet Oncol.

[74] Vascotto C (2009). Genome-wide analysis and proteomic studies reveal APE1/Ref-1 multifunctional role in mammalian cells. Proteomics.

[75] Illuzzi JL (2017). Tumor-associated APE1 variant exhibits reduced complementation efficiency but does not promote cancer cell phenotypes. Environ Mol Mutagen.

[76] Amanakis G, Murphy E (2020). Cyclophilin D: an integrator of mitochondrial function. Front Physiol.

[77] Chiu HY, Tay EXY, Ong DST, Taneja R (2020). Mitochondrial dysfunction at the center of cancer therapy. Antioxid Redox Signal.

[78] Martinez-Outschoorn UE (2011). Energy transfer in “parasitic” cancer metabolism: mitochondria are the powerhouse and Achilles’ heel of tumor cells. Cell Cycle.

[79] Martinez-Outschoorn UE, Lisanti MP, Sotgia F (2014). Catabolic cancer-associated fibroblasts transfer energy and biomass to anabolic cancer cells, fueling tumor growth. Semin Cancer Biol.

[80] Martinez-Outschoorn UE (2010). Autophagy in cancer associated fibroblasts promotes tumor cell survival: Role of hypoxia, HIF1 induction and NFkappaB activation in the tumor stromal microenvironment. Cell Cycle.

[81] Fiaschi T (2012). Reciprocal metabolic reprogramming through lactate shuttle coordinately influences tumor-stroma interplay. Cancer Res.

[82] Rhim AD (2014). Stromal elements act to restrain, rather than support, pancreatic ductal adenocarcinoma. Cancer Cell.

[83] Ozdemir BC (2014). Depletion of carcinoma-associated fibroblasts and fibrosis induces immunosuppression and accelerates pancreas cancer with reduced survival. Cancer Cell.

[84] Jiang H (2020). Pancreatic ductal adenocarcinoma progression is restrained by stromal matrix. J Clin Invest.

[85] Chun KS, Jang JH, Kim DH. Perspectives regarding the intersections between STAT3 and oxidative metabolism in cancer. Cells. 2020;9. 10.3390/cells9102202. 10.3390/cells9102202 PMC7600636 33003453

[86] Smolkova K (2020). Nuclear factor erythroid 2-related factor 2 in regulating cancer metabolism. Antioxid Redox Signal.

[87] Bazzani V (2020). Mitochondrial apurinic/apyrimidinic endonuclease 1 enhances mtDNA repair contributing to cell proliferation and mitochondrial integrity in early stages of hepatocellular carcinoma. BMC Cancer.

[88] Barchiesi A, Wasilewski M, Chacinska A, Tell G, Vascotto C (2015). Mitochondrial translocation of APE1 relies on the MIA pathway. Nucleic Acids Res.

[89] Wang X (2019). APE1/Ref-1 regulates 5-FU resistance in colon cancer cells through its redox and endonuclease activity. Int J Clin Exp Med.

