# Supplementary material for: Ref-1 redox activity alters cancer cell metabolism in pancreatic cancer: exploiting this novel finding as a potential target
Source: J Exp Clin Cancer Res. 2021 Aug 10;40:251. doi: 10.1186/s13046-021-02046-x (PMC8353735; doi:10.1186/s13046-021-02046-x)
Supplement: Supplementary file 1 — Additional file 1: Supplemental Methods. Supplementary Figure 1. Interrogation of gene and protein expression following Ref-1 inhibition. Supplementary Figure 2. Ref-1 downregulates growth of 3D spheroids in low glucose containing media conditions. Supplementary Figure 3. Ref-1 genetic or pharmacological inhibition reduces TCA cycle substrates. Supplementary Figure 4. IHC markers (vimentin and CA9) on tumors after treatment with APX2009 or Devimistat. Supplementary Figure 5. Ref-1 inhibition with additional APX analogs in combination with Devimistat attenuates growth in two co-culture models of pancreatic cancer: 3D spheroids and i-TMOC. Supplementary Figure 6. Ref-1 inhibition in combination with Devimistat shifts metabolism significantly compared to single agents. Supplemental Table 1. TMT labels used for each sample. Supplemental Table 2. qPCR Primers used for qRT-PCR. Supplemental Table 3. Complete lists of the dysregulated genes and proteins. Supplemental Table 4. Complete lists of the differentially expressed pathways. Supplemental Table 5. Predicted transcriptional regulatory factors of each identified module. Supplemental Table 6. Combination index (CI) values for the 3D assay with APX2009 in combination with Devimistat. [file 13046_2021_2046_MOESM1_ESM.docx]

Supplementary information

Supplemental Methods

Quantitative global proteomic comparison of protein levels

Cell and tissue preparation.

Cells were lysed in 8M urea, 50 mM Tris-HCl pH 8.5 (25 μL). All samples were sonicated briefly by probe sonicator, and centrifuged at 12000 rpm for 15 min to remove debris. Protein concentrations were determined using a Bradford protein assay (BioRad) and colorimeter—EPOCH|2 (BioTek Instruments, Inc., Winooski, VT 05404-0998, U.S.A.) employing vendor provided protocols. Protein samples in equal amounts were reduced with 5 mM tris(2-carboxyethyl)phosphine hydrochloride (TCEP) and alkylated with 10 mM chloroacetaminde (CAM). Samples were diluted with 100 mM Tris-HCl to a final urea concentration of 2M and digested overnight with Trypsin/Lys-C Mix Mass Spectrometry (1:100 protease:substrate ratio, Promega Corporation, Madison, WI 53711-5399, U.S.A.).

Peptide purification and labeling

Peptides were desalted on 50 mg Sep-Pak® Vac employing a vacuum manifold (Waters Corporation Milford, MA). After elution from the column in 70% ACN, 0.1% FA, peptides were dried by speed vacuum and resuspended in 24 µL of 50mM triethylammonium bicarbonate (TEAB). Peptide concentration was measured using Pierce Quantitative Colorimetric Peptide Assay Kit (Thermo Scientific) to ensure that an equal amount of each was labeled.

Samples were then Tandem Mass Tag (TMT) labeled with 0.2 mg of reagent for two hours at room temperature (11-plex kit, manufactures instructions TMT10plex™ Isobaric Label Reagent Set, Supplemental Table 1). Labelling reactions were quenched with hydroxylamine at room temperature 15 minutes. Each set of labelled peptides were then mixed and dried by speed vacuum.

High pH basic fractionation

Each peptide mixture was resuspended in 0.1% TFA (trifluoroacetic acid) and fractionated into 8 fractions on Pierce^TM^ High pH reversed-phase peptide fractionation spin columns using vendor methodology (Cat 84868). Each fraction was dried by speed vacuum and resuspended in 0.1% formic acid (FA).

Nano-LC-MS/MS Analysis

Nano-LC-MS/MS analyses were performed on an EASY-nLC™ HPLC system (Thermo Scientific) coupled to an Orbitrap Fusion™ Lumos™ mass spectrometer (Thermo Fisher Scientific). One third of each fraction was loaded onto a reversed phase PepMap^TM^ RSLC C18 column (2 μm, 100 Å, 75 μm x 25 cm) with Easy-Spray tip at 450 nL/min. Peptides were eluted from 8-20% B over 165 minutes, 30-50% B over 3 mins, 50% B for 12 minutes (Mobile phases A: 0.1% FA, water; B: 0.1% FA, 80% Acetonitrile). Mass spectrometer settings include capillary temperature of 275 ^o^C and ion spray voltage was kept at 2.6 kV. The mass spectrometer method was operated in positive ion with a 4 sec cycle time data-dependent acquisition method with advanced peak determination and Easy-IC (internal calibrant). Precursor scans (m/z 375-1500) were done with an Orbitrap resolution of 120000, RF lens% 30, maximum inject time 50 ms, 75% normalized AGC target, including charges of 2 to 7 for fragmentation with an intensity threshold of 5e3, and 60 s dynamic exclusion. CID MS2 scans were performed in the ion trap at turbo speed with a 0.7 m/z isolation window, 35% normalized CID collision energy, and an isolation window of 400-1200 m/z. 5 ions were then selected for synchronous-precursor-selection (SPS)-MS3 fragmentation with a 2 m/z window, 65% normalized HCD, Orbitrap resolution of 50000, 200% normalized AGC target and auto mode maximum IT. The data were recorded using Thermo Scientific Xcalibur software (Copyright 2017 Thermo Fisher Scientific Inc.).

Proteomic Data Analysis

Resulting RAW files were analyzed in Proteome Discover™ 2.2 (ThermoScientific) with a human uniprot FASTA database (downloaded 02/15/2017) including common contaminants. SEQUEST HT searches were conducted with a maximum number of 2 missed cleavages; precursor mass tolerance of 10 ppm; and a fragment mass tolerance of 0.8 Da. Static modifications used for the search were, 1) carbamidomethylation on cysteine (C) residues; 2) TMT 6plex label on lysine (K) residues and the N-termini of peptides. Dynamic modifications used for the search were oxidation of methionines and acetylation of N-termini. Percolator False Discovery Rate was set to a strict setting of 0.01 and a relaxed setting of 0.05. Values from both unique and razor peptides were used for quantification. In the consensus workflow, peptides were normalized in by total peptide amount and scaled on control channel 126 which was a mix of proteins from untreated cells. Resulting grouped abundance values for each sample type, abundance ratio values; and respective p-values (ANOVA) from Proteome Discover™ were exported to Microsoft Excel. The mass spectrometry proteomics data have been deposited to the ProteomeXchange Consortium via the PRIDE partner repository with the dataset identifier PXD020515 and 10.6019/PXD020515. The TMT labels fort he samples are provided in Supplementary Table 1.

Interstitial tumor-microenvironment-on-chip (iT-MOC) Assay

Cell growth was determined by quantifying the fluorescent cell area. Fluorescence of transfected Panc10.05 (TdTomato) and CAF19 (GFP) was measured with an inverted microscope (Olympus IX71, Japan) with TRITC and FITC filter on days 0, 2, 5, 8, and 9. Cell growth was analyzed by normalizing the corresponding fluorescent pixel counting by that of day 0.

$$\text{Cell growth}=\frac{[\text{Fluorescent area of cells}]_{\text{ DAY }n}\text{ } }{[\text{Fluorescent area of cells}]_{\text{ DAY0}}}\text{ }(n=0,2,5,8,and\text{ 9)}$$

Cell survival was determined by evaluating cell viability at the end of the incubations. Cell survival was defined as the cell growth at day 9 of drug treatment groups normalized by the cell growth of the control groups (absence of drug) as following:

$$\text{Cell survival}=\frac{[\text{Cell growth}]_{treatment}\text{ } }{[\text{Cell growth}]_{control}}$$

Supplemental Figure Legends

**Supplemental Figure 1: Interrogation of gene and protein expression following Ref-1 inhibition. A-D**. Validation of selected glycolysis and TCA cycle genes from the scRNA-seq data using qRT-PCR in Pa02C and Panc10.05 cells (Scr/siRef-1 – 30nM, 1% hypoxia for 24 h, n=3 for Pa02C, n=2 for Panc10.05, p<0.05-0.0001). **E-G.** Densitometry graphs for western blot images shown in Figure 3D for Pa03C, Pa02C, and Panc10.05 cells. Fold changes are normalized to SCR normoxia. **H-M.** Expression of glycolysis and TCA cycle genes after treatment with Ref-1 redox inhibitor (APX2009-10µM for Pa03C, 15µM for Pa02C, and 20µM for Panc10.05 cells for 28h) under normoxia and hypoxia (1%O_2_ for 24h) (n=2, p<0.05-0.0001). **N**. Expression of Ref-1 regulated genes after treatment with Ref-1 redox inhibitor, APX2009 at 10µM under normoxia and hypoxia (1% O_2_ for 24h) in Pa03C cells (n=2, p<0.05-0.0001). **O.** Ref-1 regulated gene panel after treatment with  Ref-1 redox inhibitor (APX2009, 5µM)  compared to vehicle control (DMSO) in Pa03C 3D spheroids (n=3, p<0.05-0.0001) with BIRC5 (survivin) and CA9 as positive controls for Ref-1 inhibition and hypoxia induction.

**Supplemental Figure 2: Ref-1 downregulates growth of 3D spheroids regardless of media conditions.** Pa03C cells alone (**A**) or as co-cultures with CAF19 (**B**) were plated as 3D spheres after transfection with Scr or siRef-1 siRNA and were grown in the presence of complete growth media or low glucose media. Intensity of fluorescence was measured on days 4, 8, and 12. (n≥3, Mean ± S.E. p<0.05-0.0001).

**Supplemental Figure 3: Ref-1 genetic or pharmacological inhibition reduces TCA cycle substrates. A.** TCA Cycle showing its intermediates entering other biomolecule synthesis pathways. Line graphs showing kinetics for TCA cycle substrates in Pa03C cells with Scr vs 10nM siRef-1 (**B**), Ref-1 redox inhibition (**C**, APX2009), inactive Ref-1 redox inhibitor analog (**D**, RN7-58-5), metabolic inhibitor, Devimistat (**F**), and in CAF02 cells after treatment with APX2009 (**G**). Average rate of reaction in Pa03C cells treated with metabolic inhibitor Devimistat (**E**, n=3, *p<0.05, **0.01).

**Supplemental Figure 4: IHC markers on tumors after treatment with APX2009 or Devimistat.** **A**. Quantitation of IHC staining of Panc10.05+CAF xenograft tumors with Vimentin. Data represented as Mean ± SE. **B**. Representative images for CA9 IHC staining of Pa03C and Panc10.05+CAF19 tumors (Scale bar – 200 µm).

**Supplemental Figure 5: Ref-1 inhibition with additional APX analogs in combination with Devimistat attenuates growth in two co-culture models of pancreatic cancer: 3D spheroids and i-TMOC. A.** 3D co-cultures of Pa03C or Panc10.05 with CAF19 cells plated at a ratio of 1:4 and treated with DMSO or APX3330 (35 µM) or APX2014 (1 µM) or Devimistat (25 µM  Pa03C and 50 µM for Panc10.05) alone or in combination with APX3330 or APX2014. Graphs represent either tumor intensity or CAF19 intensity measured as fluorescence on Days 4, 7, 10, and 14. **B&C.** Quantitation of cell growth over time of Panc10.05+CAF19 co-cultures on iT-MOC either with the single agent (APX2009 – 30 µM/Devimistat – 25 µM) or as combination treatment (n=3). All data represent Mean±SE.

**Supplemental Figure 6: Ref-1 inhibition in combination with Devimistat shifts metabolism significantly compared to single agents.** Expression of Ref-1 regulated genes **(A)** via qPCR in Pa03C 3D spheroids treated with DMSO or a combination of APX2009 (5 µM) and Devimistat (50 µM) (n=3, p<0.05-0.0001). The data with APX2009 is also in Supplemental Figure 1O and provided here for comparison to combination. Line graphs (**B**) showing kinetics for TCA cycle substrates in Pa03C cells with the metabolic inhibitor Devimistat (50µM) in combination with APX2009 (5 and 10µM, n=3).

Supplemental Table Legends

Supplementary Table 1. TMT labels used for each sample.

Supplemental Table 2. qPCR Primers used for qRT-PCR.

Supplementary Table 3. Complete lists of the dysregulated genes and proteins.

Supplemental Table 4. Complete lists of the differentially expressed pathways.

Supplemental Table 5. Predicted transcriptional regulatory factors of each identified module.

Supplemental Table 6. Combination index (CI) values for the 3D assay with APX2009 in combination with Devimistat.

Supplementary Table 1. TMT labels used for each sample.


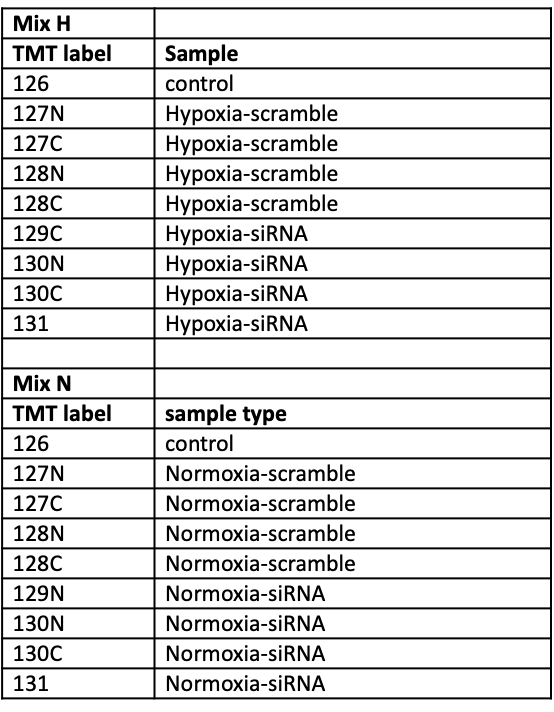


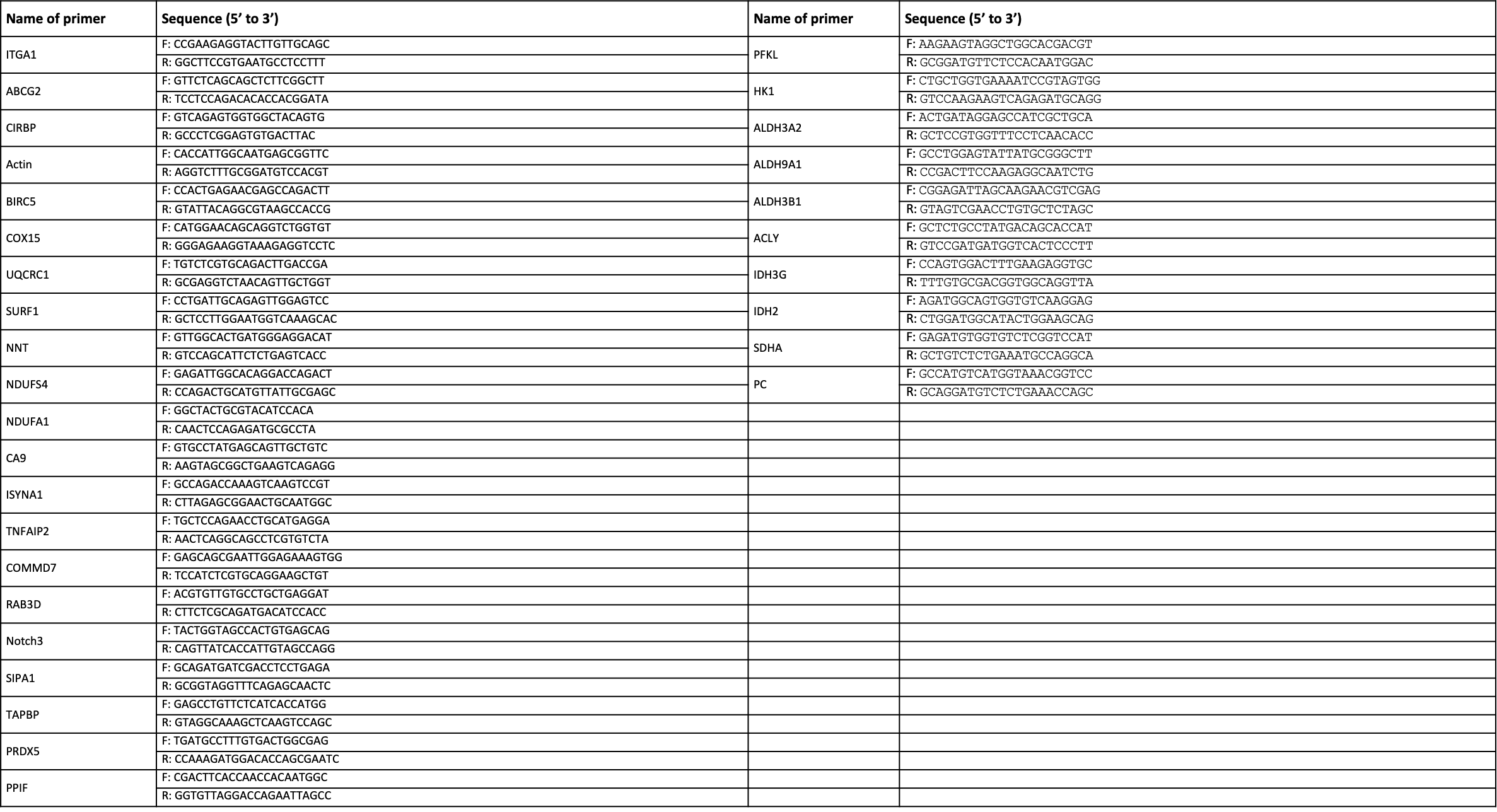


Supplemental Table 2. qPCR Primers used for qRT-PCR


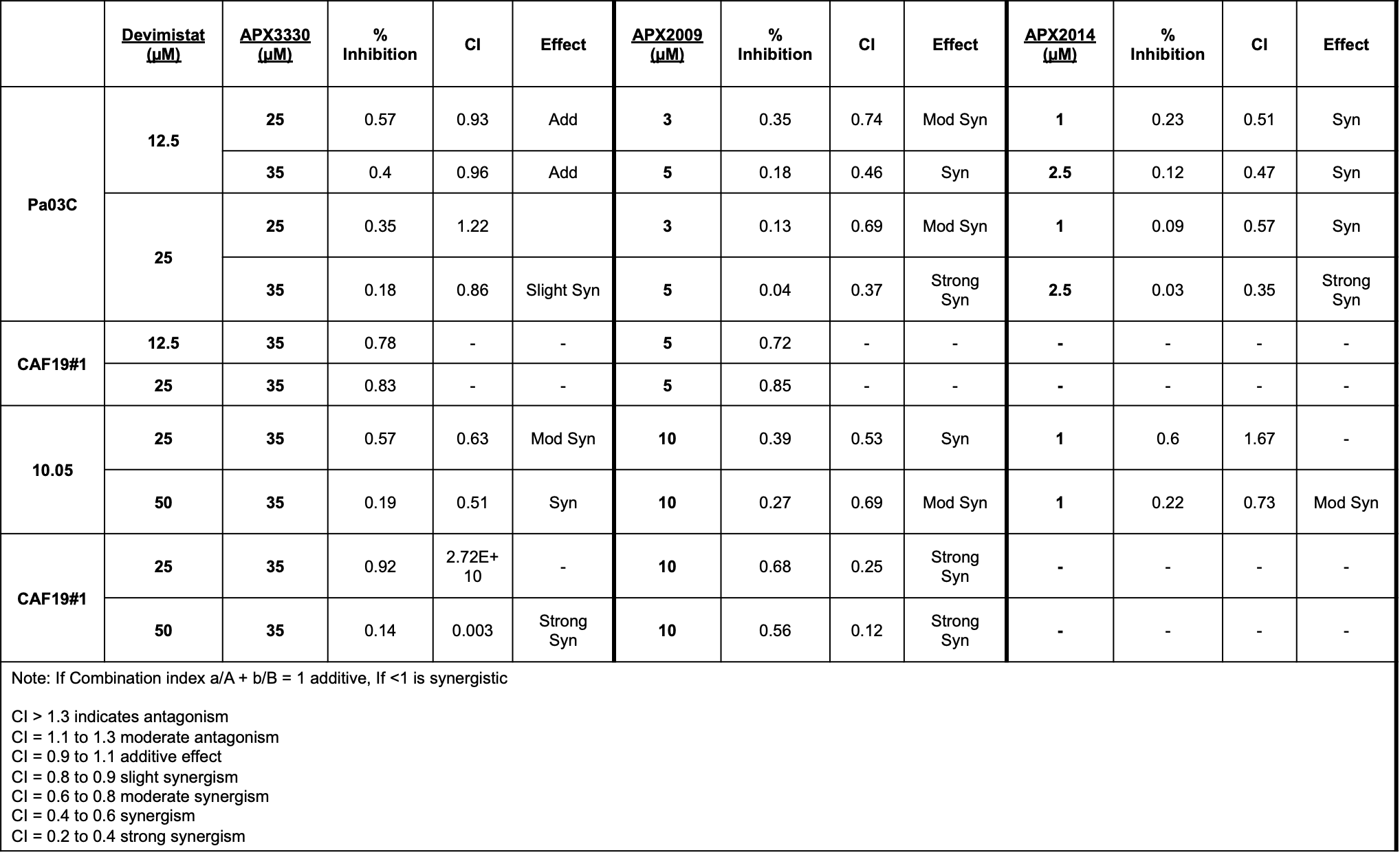


Supplemental Table 6. Combination index (CI) values
