# Supplementary material for: Ref-1 redox activity alters cancer cell metabolism in pancreatic cancer: exploiting this novel finding as a potential target
Source: J Exp Clin Cancer Res. 2021 Aug 10;40:251. doi: 10.1186/s13046-021-02046-x (PMC8353735; doi:10.1186/s13046-021-02046-x)
Supplement: Supplementary file 2 — Additional file 2. [file 13046_2021_2046_MOESM2_ESM.pptx]

## Slide 1
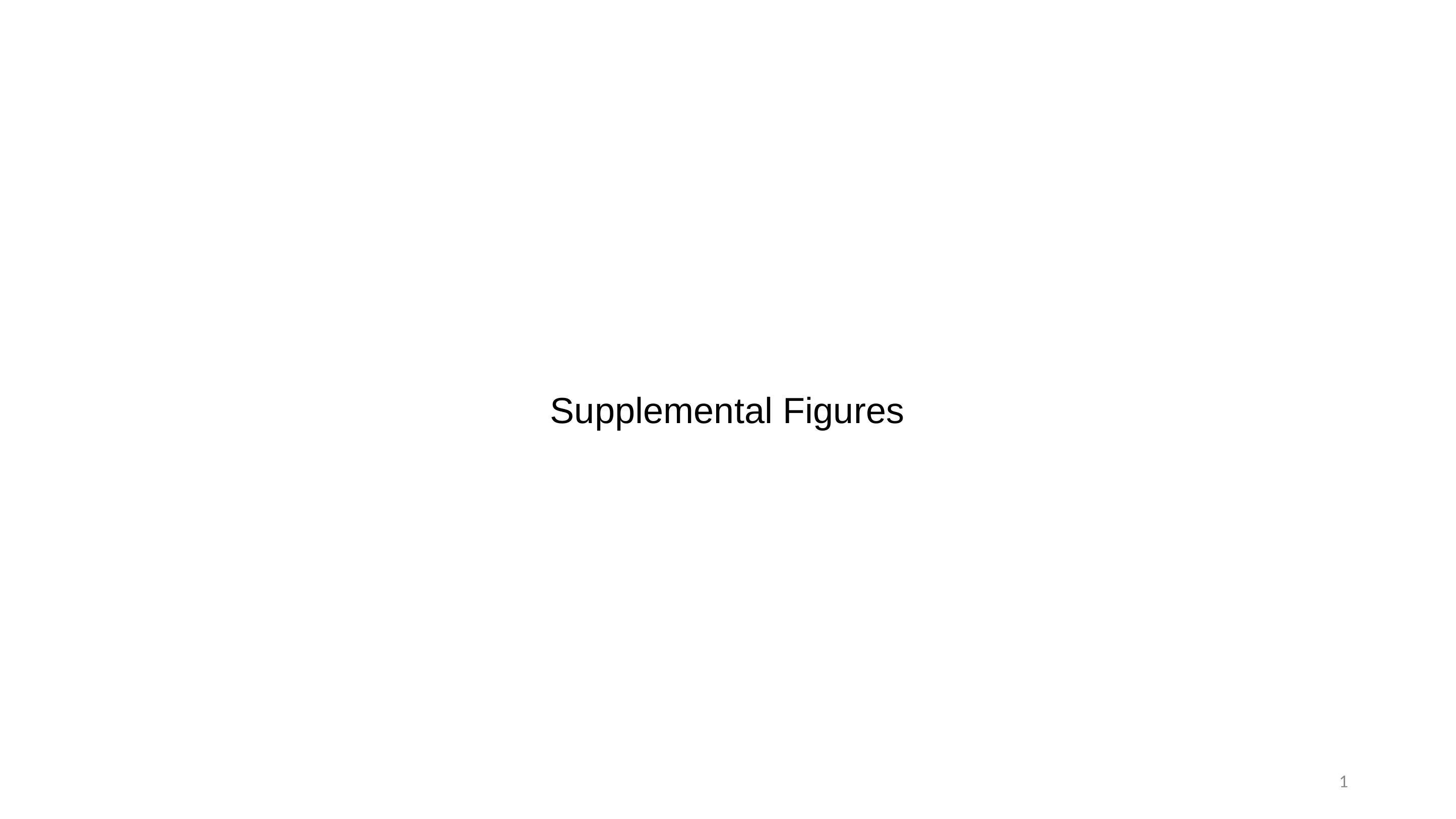

Supplemental Figures
1

## Slide 2
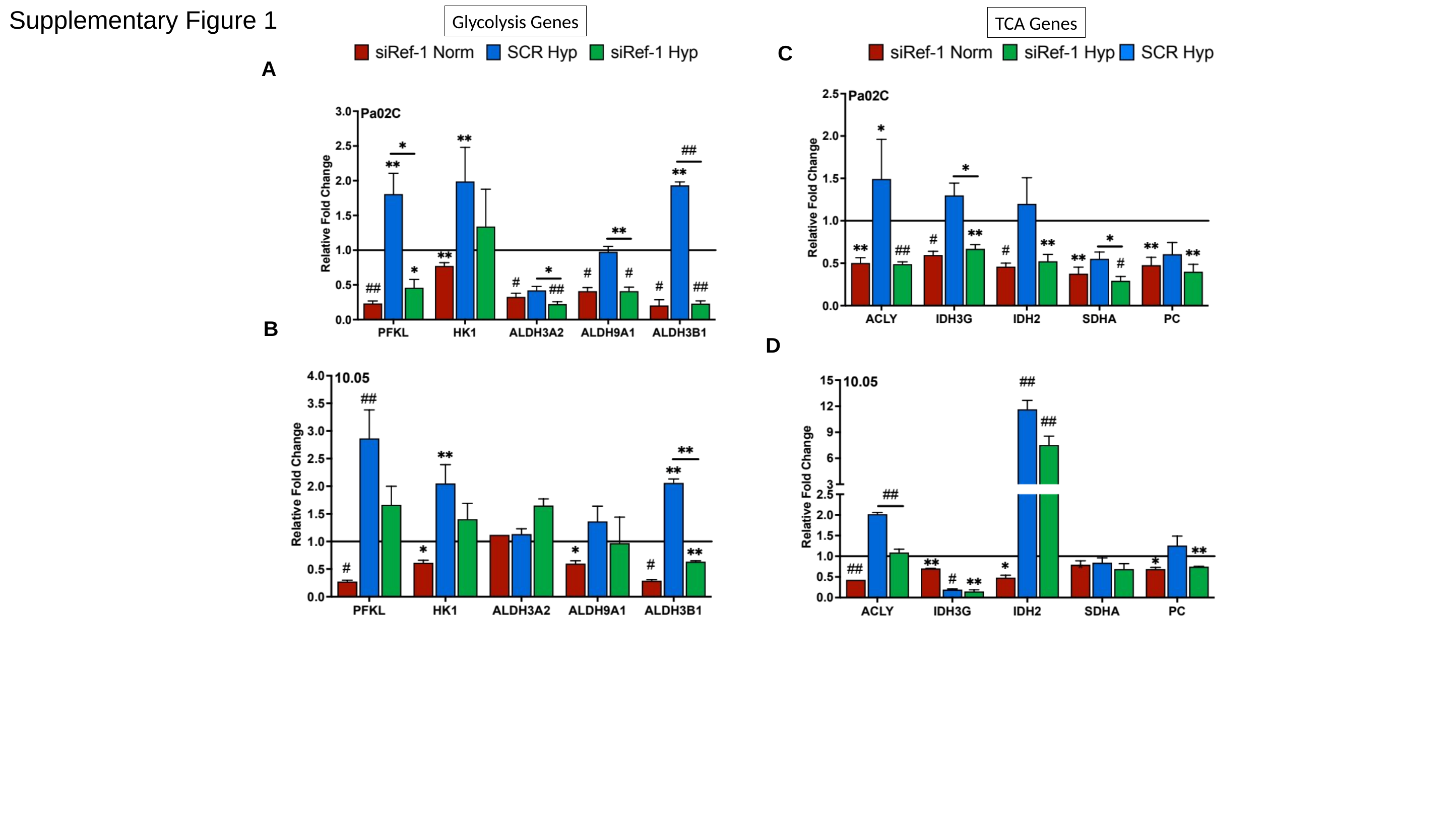

Supplementary Figure 1
Glycolysis Genes
TCA Genes
C
A
B
D

## Slide 3
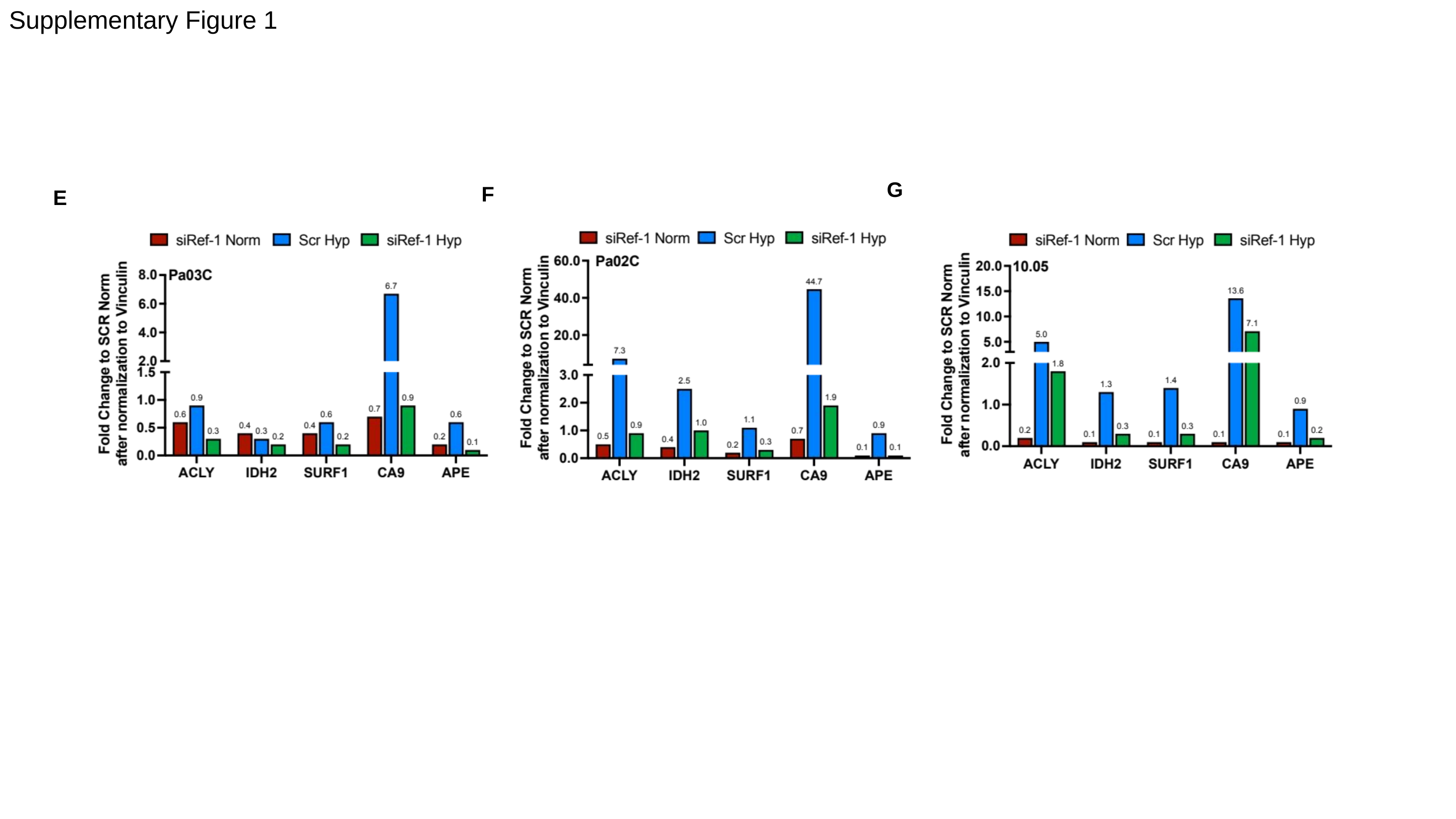

Supplementary Figure 1
G
F
E

## Slide 4
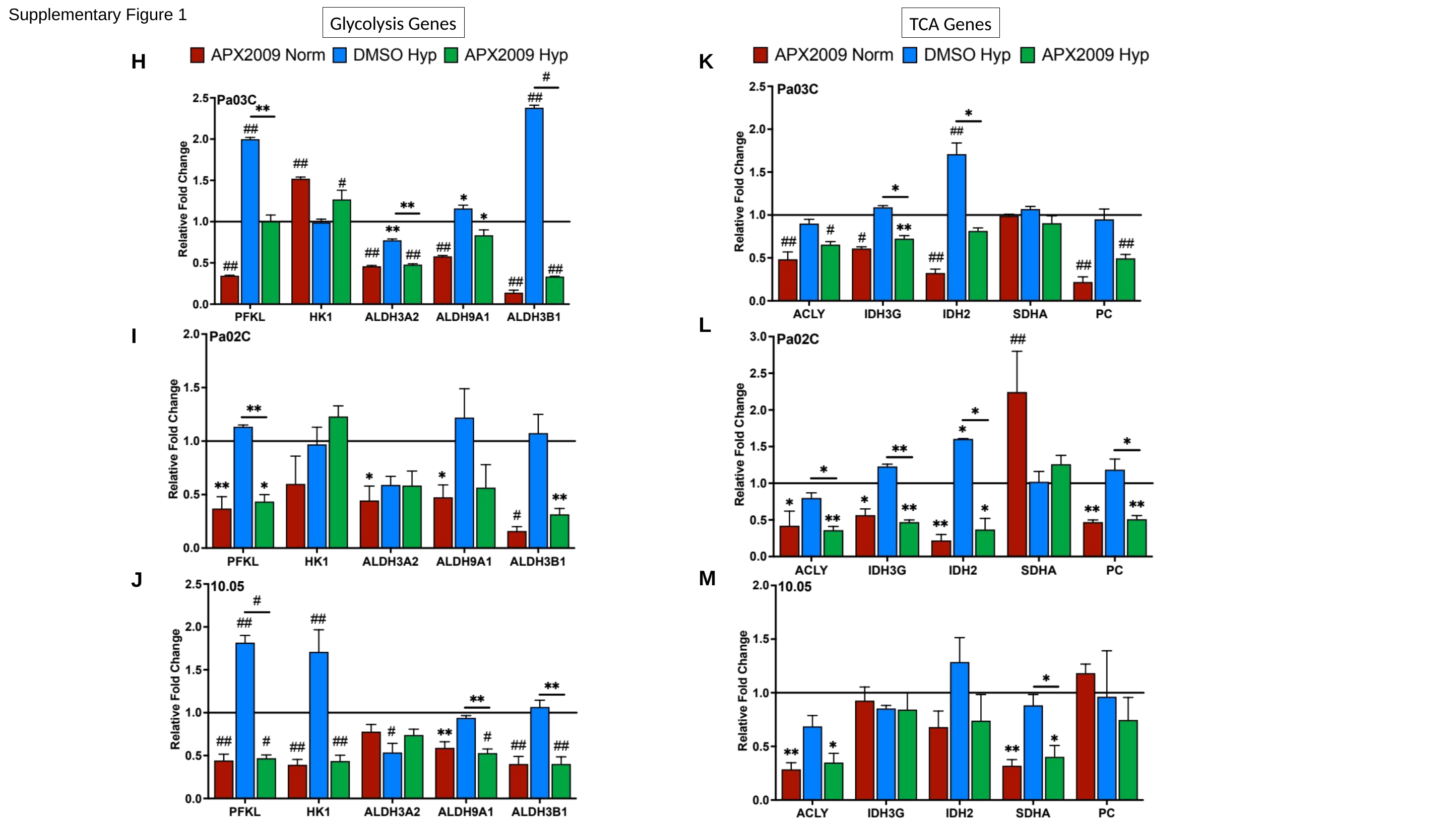

Supplementary Figure 1
Glycolysis Genes
TCA Genes
H
K
L
I
M
J

## Slide 5
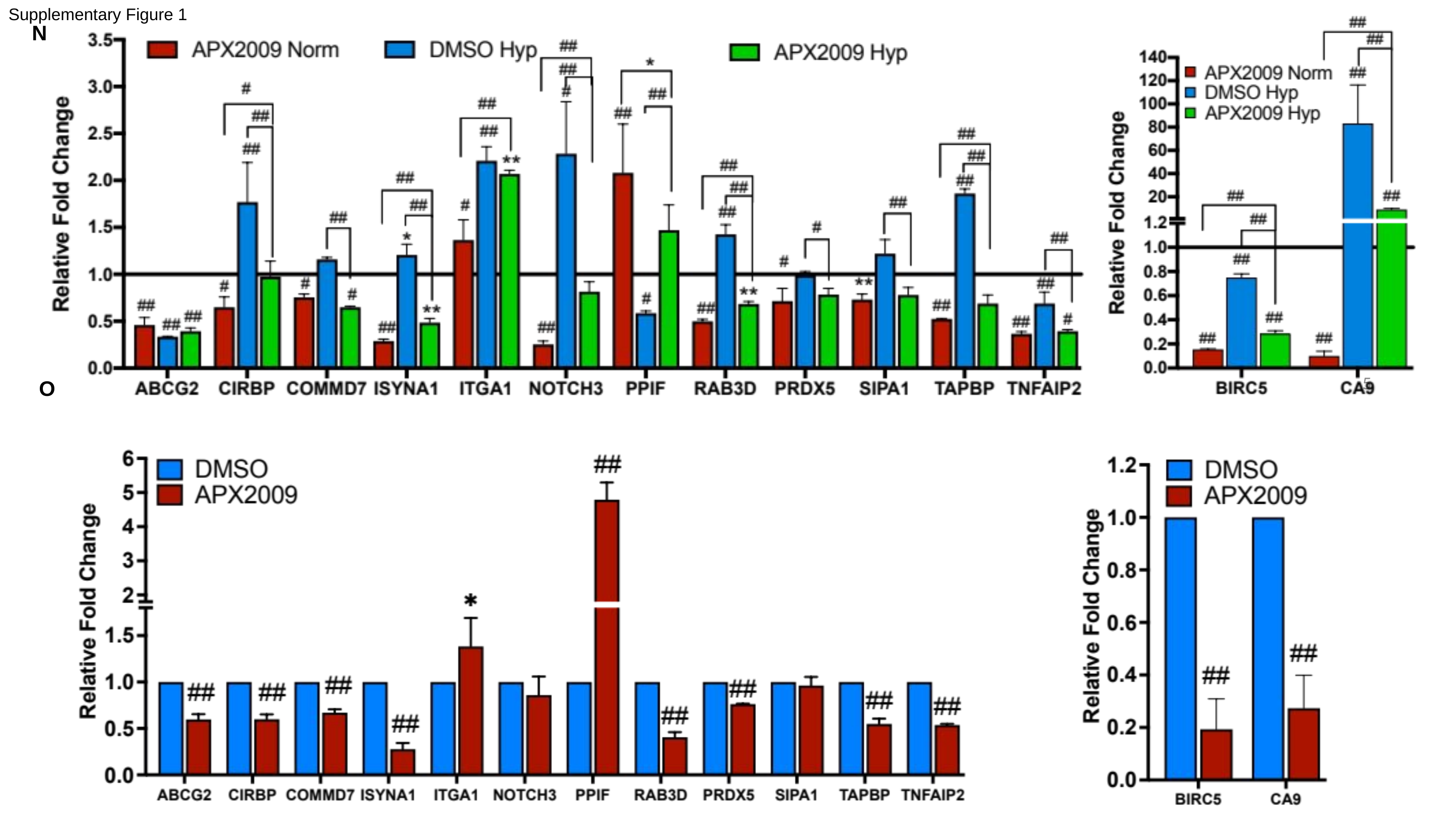

Supplementary Figure 1
N
5
O

## Slide 6
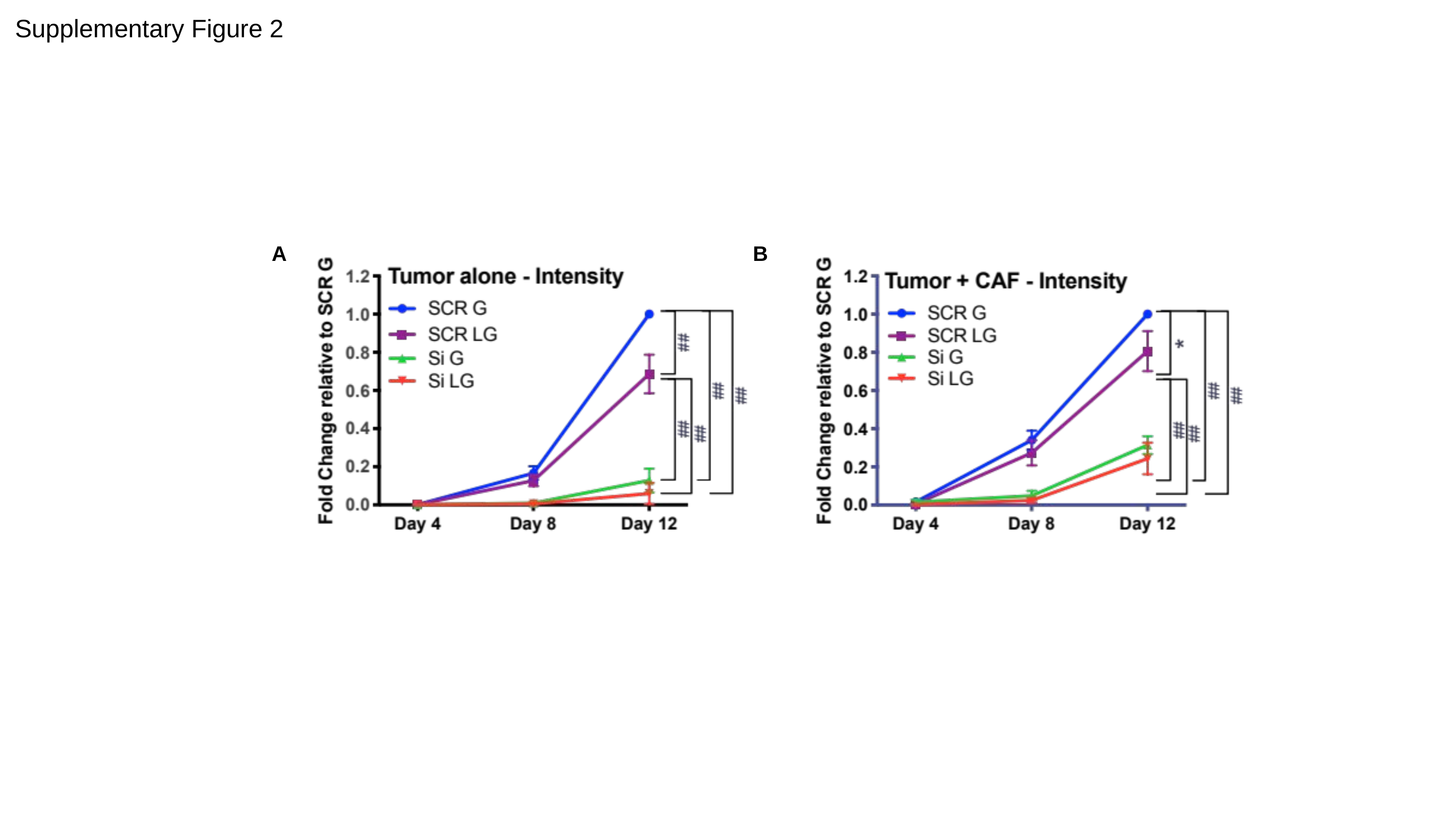

Supplementary Figure 2
A
B

## Slide 7
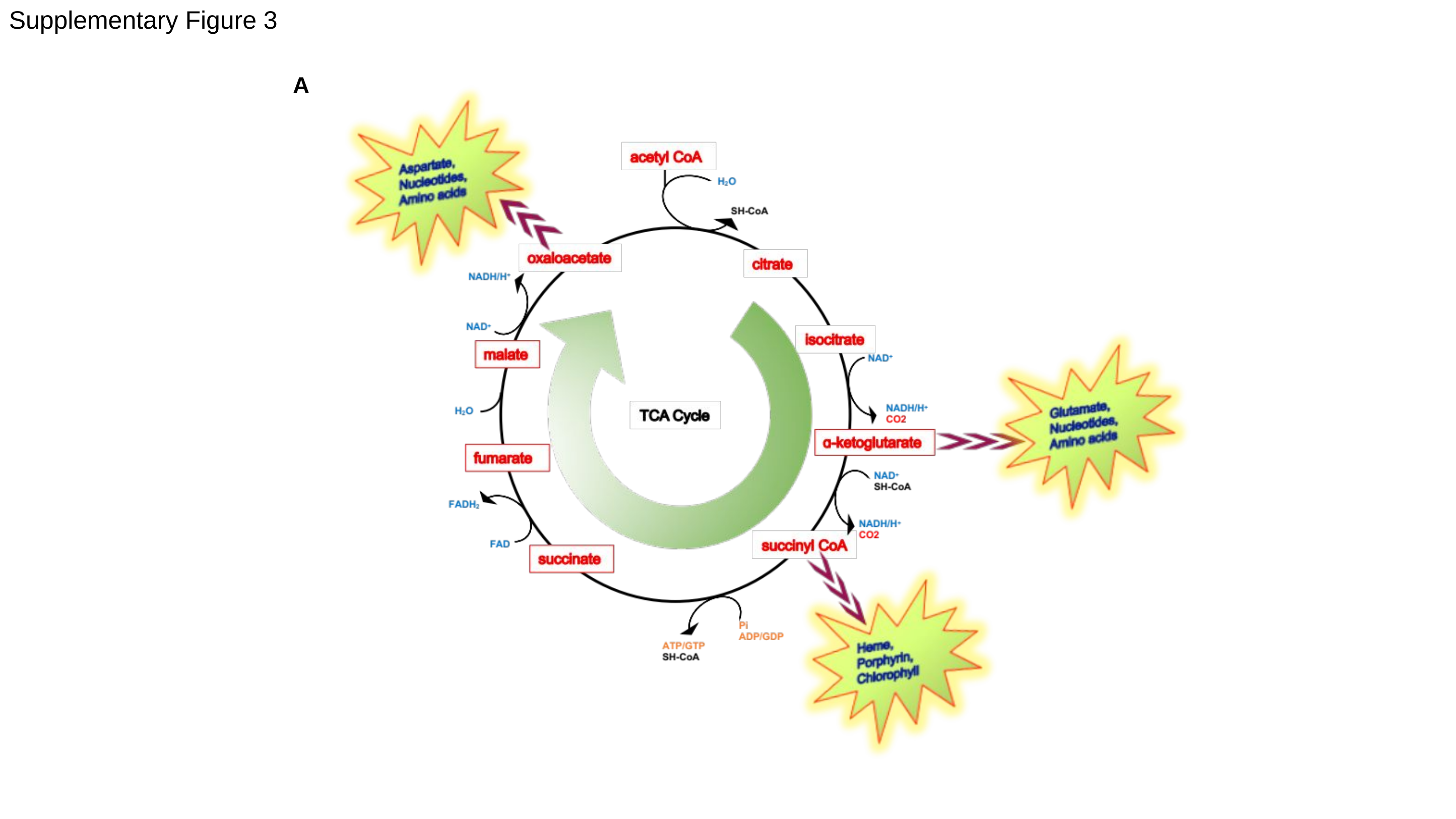

Supplementary Figure 3
A

## Slide 8
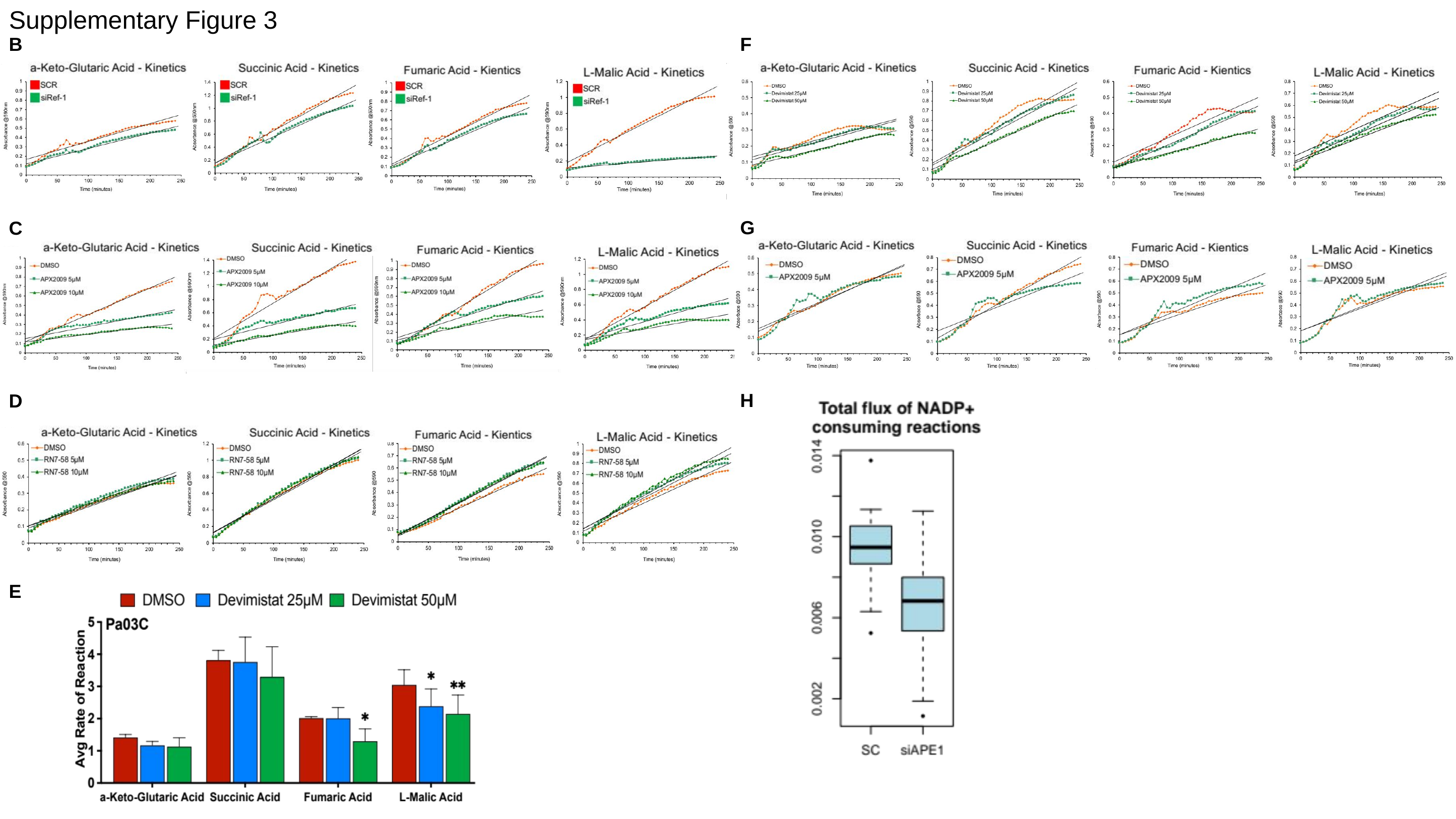

Supplementary Figure 3
B
F
G
C
H
D
E

## Slide 9
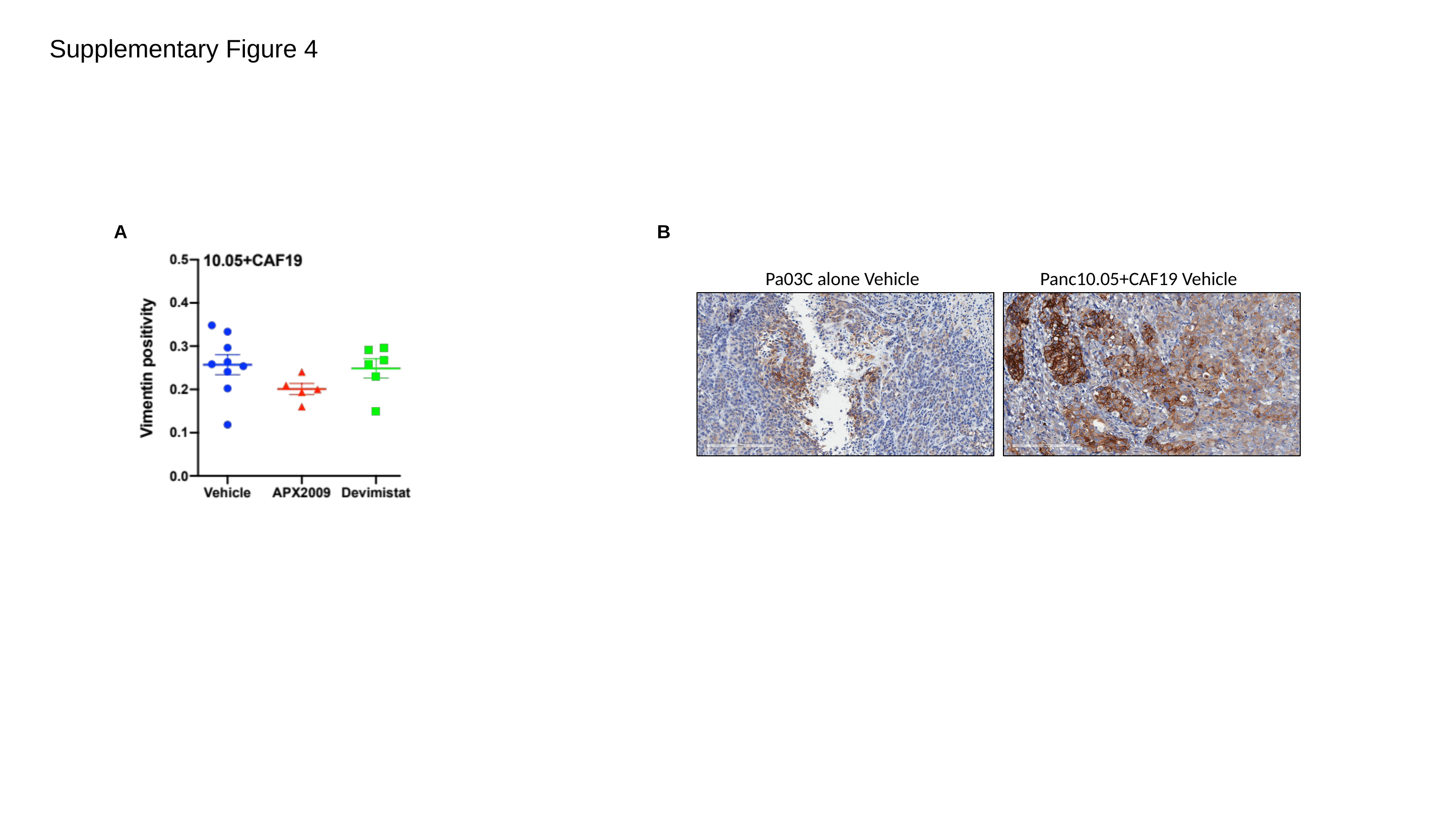

Supplementary Figure 4
A
B
Pa03C alone Vehicle
Panc10.05+CAF19 Vehicle

## Slide 10
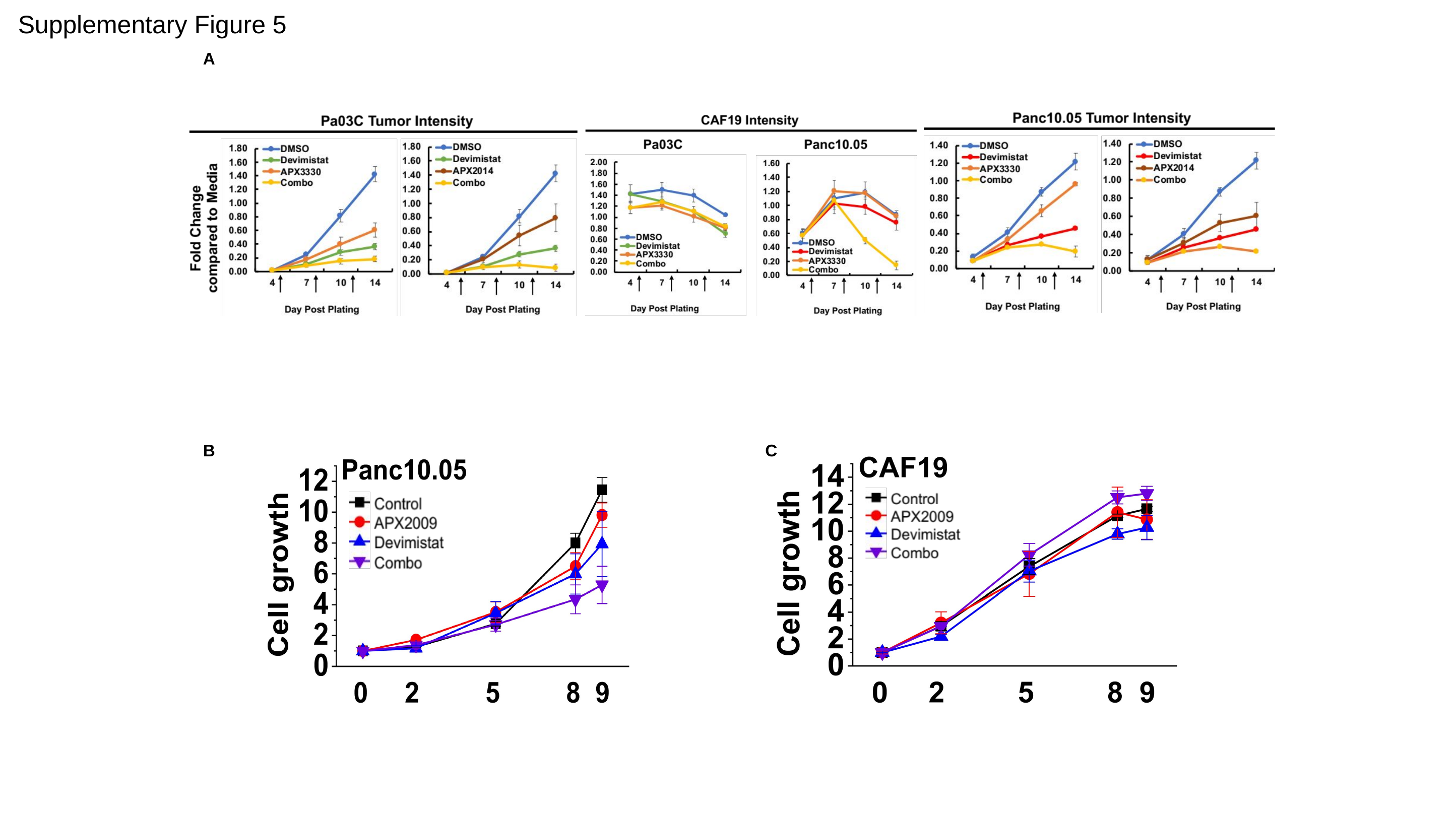

Supplementary Figure 5
A
B
C

## Slide 11
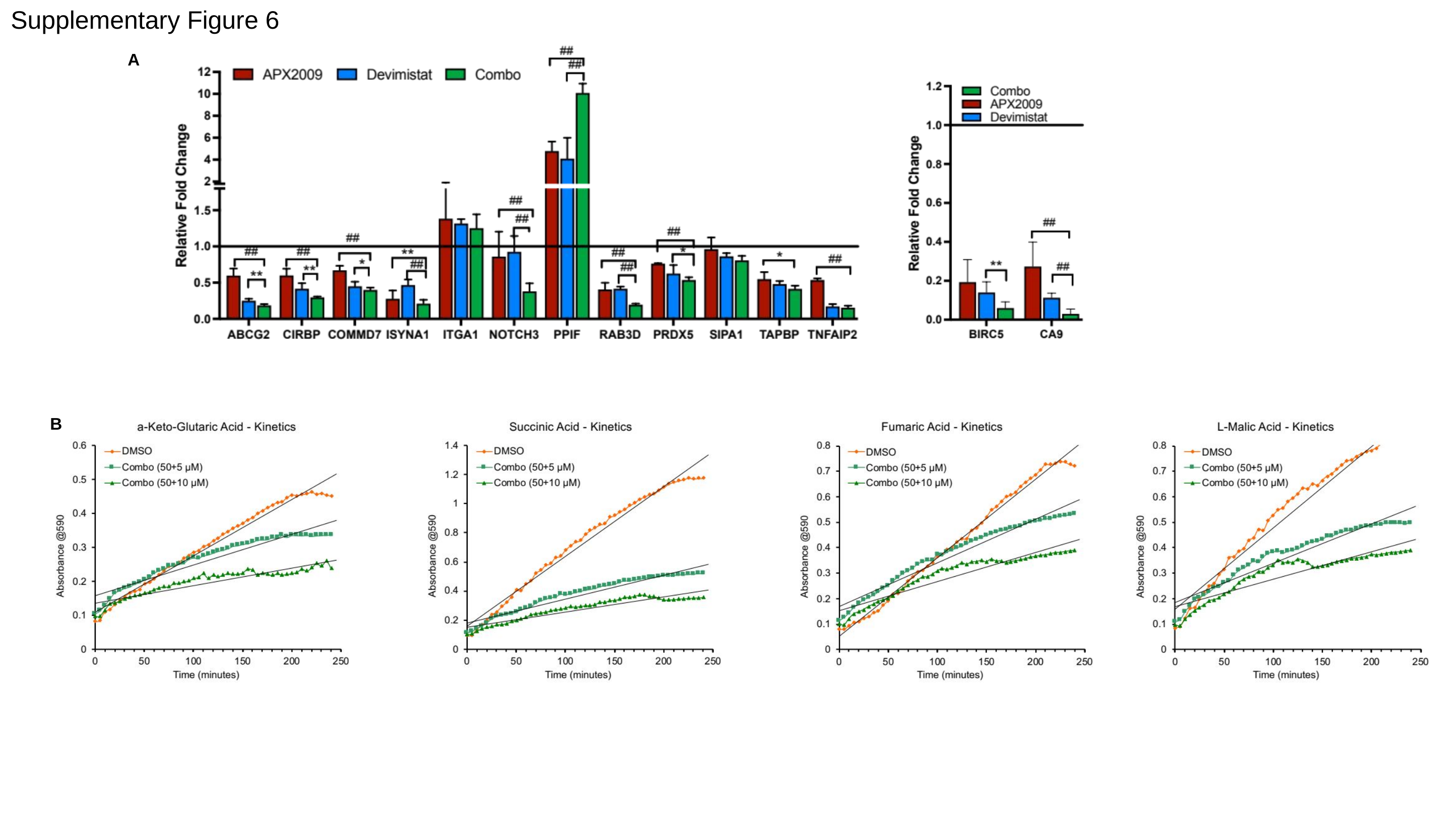

Supplementary Figure 6
A
B
